# Retinal nerve fibre layer thickness is associated with attention and predicts risk states of dementia

**DOI:** 10.1093/braincomms/fcaf464

**Published:** 2025-12-05

**Authors:** Matthias L Schroeter, Johanna Girbardt, Tobias Luck, Francisca S Rodriguez, Gordon T Plant, Barbara Wicklein, Kerstin Wirkner, Christoph Engel, Jana Kynast, Christian Girbardt, Mengyu Wang, Maryna Polyakova, Andreas Hinz, A Veronica Witte, Toralf Kirsten, Markus Loeffler, Arno Villringer, Steffi G Riedel-Heller, Tobias Elze, Franziska G Rauscher

**Affiliations:** Department of Neurology, Max Planck Institute for Human Cognitive and Brain Sciences, 04103 Leipzig, Germany; Clinic of Cognitive Neurology, Leipzig University Hospital, 04103 Leipzig, Germany; Leipzig Research Centre for Civilization Diseases (LIFE), Leipzig University, 04103 Leipzig, Germany; Department of Neurology, Max Planck Institute for Human Cognitive and Brain Sciences, 04103 Leipzig, Germany; Institute for Medical Informatics, Statistics and Epidemiology, Leipzig University, 04107 Leipzig, Germany; Faculty of Applied Social Sciences, University of Applied Sciences Erfurt, 99085 Erfurt, Germany; Institute of Social Medicine, Occupational Health and Public Health (ISAP), Leipzig University, 04103 Leipzig, Germany; German Centre for Neurodegenerative Diseases (DZNE), Research Group Psychosocial Epidemiology and Public Health, 17489 Greifswald, Germany; Institute of Neurology, University College London, WC1E 6BT London, UK; Institute for Medical Informatics, Statistics and Epidemiology, Leipzig University, 04107 Leipzig, Germany; Leipzig Research Centre for Civilization Diseases (LIFE), Leipzig University, 04103 Leipzig, Germany; Institute for Medical Informatics, Statistics and Epidemiology, Leipzig University, 04107 Leipzig, Germany; Leipzig Research Centre for Civilization Diseases (LIFE), Leipzig University, 04103 Leipzig, Germany; Institute for Medical Informatics, Statistics and Epidemiology, Leipzig University, 04107 Leipzig, Germany; Department of Neurology, Max Planck Institute for Human Cognitive and Brain Sciences, 04103 Leipzig, Germany; Department of Ophthalmology, Leipzig University Medical Center, 04103 Leipzig, Germany; Schepens Eye Research Institute, Harvard Medical School, Boston, MA 02114, USA; Department of Neurology, Max Planck Institute for Human Cognitive and Brain Sciences, 04103 Leipzig, Germany; Department for Medical Psychology and Sociology, Leipzig University Medical Center, 04103 Leipzig, Germany; Department of Neurology, Max Planck Institute for Human Cognitive and Brain Sciences, 04103 Leipzig, Germany; Leipzig Research Centre for Civilization Diseases (LIFE), Leipzig University, 04103 Leipzig, Germany; Leipzig Research Centre for Civilization Diseases (LIFE), Leipzig University, 04103 Leipzig, Germany; Institute for Medical Informatics, Statistics and Epidemiology, Leipzig University, 04107 Leipzig, Germany; Medical Informatics Center—Department of Medical Data Science, Leipzig University Medical Center, 04103 Leipzig, Germany; Leipzig Research Centre for Civilization Diseases (LIFE), Leipzig University, 04103 Leipzig, Germany; Institute for Medical Informatics, Statistics and Epidemiology, Leipzig University, 04107 Leipzig, Germany; Department of Neurology, Max Planck Institute for Human Cognitive and Brain Sciences, 04103 Leipzig, Germany; Clinic of Cognitive Neurology, Leipzig University Hospital, 04103 Leipzig, Germany; Leipzig Research Centre for Civilization Diseases (LIFE), Leipzig University, 04103 Leipzig, Germany; Leipzig Research Centre for Civilization Diseases (LIFE), Leipzig University, 04103 Leipzig, Germany; Institute of Social Medicine, Occupational Health and Public Health (ISAP), Leipzig University, 04103 Leipzig, Germany; Leipzig Research Centre for Civilization Diseases (LIFE), Leipzig University, 04103 Leipzig, Germany; Schepens Eye Research Institute, Harvard Medical School, Boston, MA 02114, USA; Leipzig Research Centre for Civilization Diseases (LIFE), Leipzig University, 04103 Leipzig, Germany; Institute for Medical Informatics, Statistics and Epidemiology, Leipzig University, 04107 Leipzig, Germany; Medical Informatics Center—Department of Medical Data Science, Leipzig University Medical Center, 04103 Leipzig, Germany

**Keywords:** retinal nerve fibre layer thickness, mild cognitive impairment, neurocognitive disorder, hippocampus, MRI

## Abstract

Alzheimer’s disease is associated with lower circumpapillary retinal nerve fibre layer thickness (cpRNFLT). It remains unclear if dementia risk states, i.e. mild cognitive impairment (MCI) and mild neurocognitive disorder (NCD) might associate with cpRNFLT and whether specific domains of cognitive function are related. The present study compared systematically all cognitive domains as defined in the Diagnostic and Statistical Manual of Mental Disorders (DSM-5) with pointwise analyses of the cpRNFLT and whether cpRNFLT variation can predict MCI and mild NCD. Spectral domain optical coherence tomography scans (768 A-scans of cpRNFLT) were analysed from 1300 participants with reliable measurements, without eye diseases, and further exclusion due to brain disorders. The study was conducted in the framework of the population-based Leipzig Research Centre for Civilization Diseases-(LIFE)-Adult study. The six DSM-5 domains were operationalized by means of both (sub-)scales of the ‘Consortium to Establish a Registry for Alzheimer Disease’ (CERAD-Plus) neuropsychological test battery and the ‘Reading the Mind in the Eyes’ test. Age, sex, education and scanning radius were used as additional regressors to adjust for demographics and eye anatomy. 2133 eyes of 1300 subjects were selected (age range 60–79 years). After adjustment for multiple comparisons, in the domain ‘attention’, worse performance was related to significantly thinner cpRNFL, especially in male participants, most pronounced for temporal and nasal-superior locations. For the domain ‘executive function’ significantly thicker cpRNFL was found nasally in female participants. There were no significant (*P* < 0.05) cpRNFLT locations for the DSM-5 domains ‘learning/memory’, ‘perceptual-motor abilities’, ‘language’ and ‘social cognition’. Subjects with MCI had thinner cpRNFL temporal-superior compared to subjects with normal cognition. Furthermore, alterations of cpRNFLT in MCI and mild NCD, and subgroups amnestic MCI and amnestic mild NCD existed, for the latter mainly in temporal regions. Compared to cognitively unimpaired, analyses revealed hippocampal volume decreases in MCI and mild NCD groups, and comparable white matter lesion volume, compatible with Alzheimer aetiology. cpRNFL fibre thinning was most prominently associated with lower performance in the attention domain. Highly location specific thinning involved predominantly retinal locations superior and temporal to the optic disc. Thinning in temporal-superior segment was associated with MCI. Temporal thinning indicated amnestic MCI and amnestic mild NCD. Executive function, MCI, and mild NCD presented a concordantly negative association of cognition and RNFLT nasally. As cpRNFLT is obtained conveniently within seconds, our results might assist clinicians by earlier identification of patients at risk for developing cognitive decline associated with diseases like Alzheimer’s disease.

## Teaser

Schroeter and Girbardt *et al*. provide insight into the relationship between peripapillary retinal nerve fibre layer thickness (cpRNFLT) and performance in multiple cognitive domains as markers of early cognitive decline. Alterations of cpRNFLT are localized to specific retinal sectors and show correlations which are sex and cognitive domain specific

## Graphical Abstract

**Graphical Abstract:**
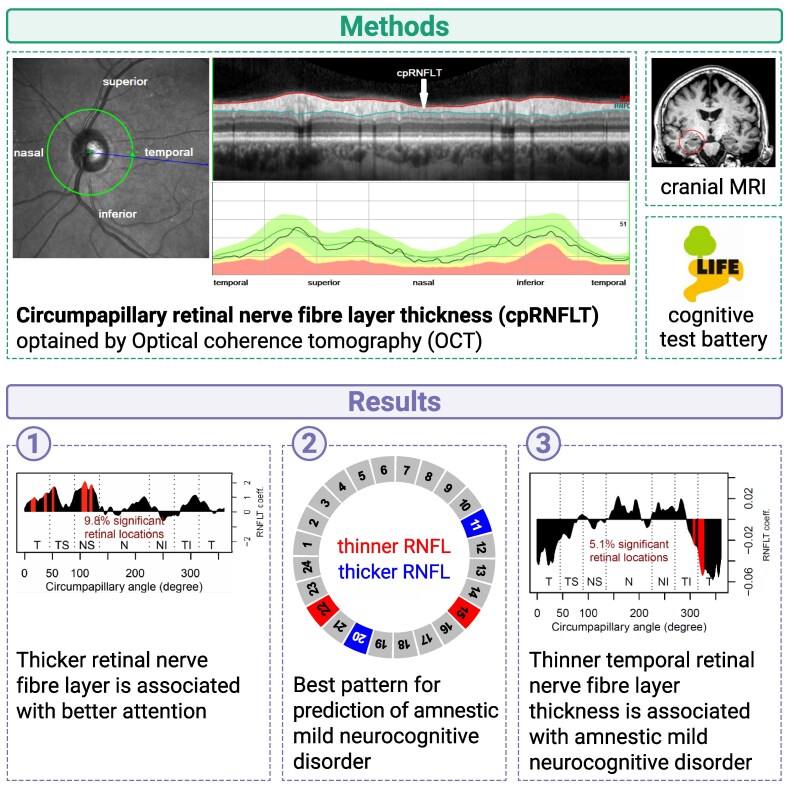

## Introduction

Retinal nerve fibre loss has previously been associated with impaired cognitive performance^1^ and dementia, in particular in Alzheimer’s disease (AD).^2^ Notably, the cerebral cortex and retina, directly interconnected, share embryologic origin, neuronal cell layers, and complex neurotransmitter systems.^3^ Imaging of the brain requires sophisticated methods, such as magnetic resonance imaging (MRI), but the retina can be rapidly and easily assessed with ophthalmic imaging techniques. Accordingly, retinal imaging represents a promising tool to detect early changes in cognition.

Pathophysiologically, several explaining mechanisms simultaneous changes in the brain and eye are under consideration: some studies have shown evidence that brain lesions can cause changes in retinal nerve fibre layer thickness (RNFLT) due to retrograde transsynaptic degeneration;^4-6^ furthermore, joint brain/eye—alterations due to vascular pathology^7^ or local amyloid plaques^8,9^ can play a role, for example.

Recently, diagnostic approaches have been refocused to include risk states of dementia to enable early therapeutic intervention, for example, to remove pathogenic proteins such as amyloid.^10,11^ In relation to this, the category ‘Mild cognitive impairment’ (MCI) was introduced, in particular its amnestic subtype representing a risk state for AD.^12,13^ Note that MCI also might be related to other aetiologies besides AD, in particular psychiatric diseases such as depression^14^ and metabolic diseases.^15,16^ Mild neurocognitive disorder (NCD)—as defined according to the Diagnostic and Statistical Manual of Mental Disorders (DSM)-5—broadened this concept by including dementia syndromes other than AD.^17^ Both, MCI and mild NCD, are characterized by cognitive decline from a previous level of performance as recognized by the participant; by a decline in objective cognitive performance detected by standardized neuropsychological testing; and by preserved independence in everyday activities in contrast to the more severe stages, namely dementia or major NCD.

Concerning risk states of dementia, there is meta-analytical evidence for a thinner circumpapillary retinal nerve fibre layer thickness (cpRNFLT) in MCI subjects versus controls, which is less pronounced than in AD.^18^ However, results are still inconsistent in what has been studied involving relatively few participants. For example, Knoll *et al*. found thicker cpRNFL to be associated with worse cognition^19^ while others found no alterations of cpRNFLT.^20-22^ A population-based Japanese survey also found no significant changes in cpRNFLT in MCI patients.^23^

A few investigations have addressed the possibility of changes in cpRNFLT in association with isolated cognitive dimensions, but again with heterogeneous results. In a case–control study with 24 MCI patients, the inferior quadrant was correlated with memory impairment.^24^ Another study with 42 Ad and 26 amnestic MCI participants found no correlation with single cognitive domains.^25^ An investigation of 20 healthy older adults revealed a positive correlation of thicker specific cpRNFL sectors with better performance on tests of working memory, psychomotor speed, and executive function.^26^ In the population-based Rhineland-Study (2483 participants), a non-significant trend of thinner global cpRNFLT was shown for all investigated cognitive domains; furthermore, changes in macular ganglion cell layer thickness presented statistically significant changes with cognition.^27^ In the UK Biobank population study, thinner macular retinal nerve fibre layer (RNFL) was also associated with worse cognition.^28^ As macular retinal nerve fibres represent only a part of the potential information of RNFL throughout the eye (matching to temporal and temporal-inferior peripapillary segments), we chose to analyse the peripapillary RNFL as it represents all retinal nerve fibres of the fundus. Other population-based studies (e.g. Northern Ireland Cohort for the Longitudinal Study of Ageing (NICOLA), Three-City-Alienor longitudinal population-based cohort, the Lothian Birth Cohort 1936, amongst others) demonstrated important findings in this peripapillary area. With our high-resolution optical coherence tomography (OCT) analysis, we examine cpRNFL in unprecedented detail.

In the present study, we associated systematically all cognitive domains as defined in the DSM-5 with high-resolution cpRNFLT (768 A-Scans, referred to as ‘locations’ in the text). Taking all cognitive domains into account enabled us to investigate any correlation of the measures with a range of dementia syndromes, further to memory-centred studies focusing on AD only. Additionally, we analysed whether variations in cpRNFLT can predict ‘risk states’ for dementia, i.e. MCI and mild NCD. Spectral domain optical coherence tomography (SD-OCT) scans were analysed in a large population-based study of 1300 participants. The six DSM-5 domains were operationalized by (sub-)scales of the Consortium to Establish a Registry for AD (CERAD-Plus) neuropsychological test battery^29,30^ and the Reading the Mind in the Eyes Test.^31^ We hypothesized that impairment in cognitive domains is associated with location-specific alteration of cpRNFLT and that these alterations of cpRNFLT might predict MCI and mild NCD.

## Materials and methods

### Participants

**Figure 1:**
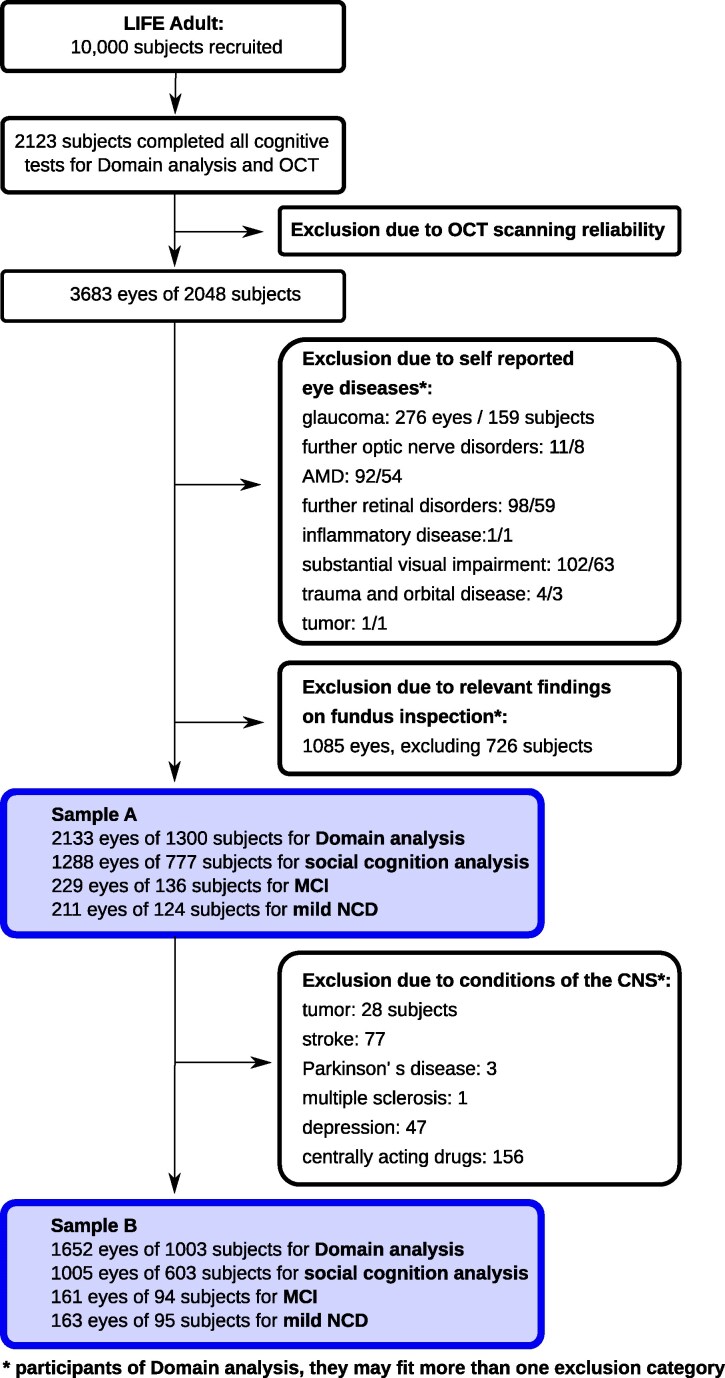
**Flowchart illustrating inclusion and exclusion of participants.** After exclusion of data based on image quality and ocular conditions, sample A consisted of 2133 eyes for domain analysis; the subpopulation of ‘social cognition’ comprises 1288 eyes; 229 eyes of participants with mild cognitive impairment (MCI) and 211 eyes of participants with mild neurocognitive disorder (NCD) were analysed. To account for CNS confounders, we additionally excluded the following conditions: tumour, stroke, Parkinson’s disease, multiple sclerosis, depression and intake of centrally acting drugs. This strictly controlled data set is labelled sample B, including 1652 eyes for domain analysis, 1005 for social cognition analysis, 161 eyes of participants with MCI-diagnosis and 163 with mild NCD diagnosis. AMD, age-related macular degeneration; OCT, optical coherence tomography; CNS, central nervous system.

Data were obtained from the population-based LIFE-Adult-Study conducted by the Leipzig Research Center for Civilization Diseases (LIFE) in Leipzig, Germany. The study design has been described in detail elsewhere.^32,33^ The study included overall 10 000 age- and sex-stratified (female, male) residents of the city of Leipzig, Germany, with a main focus on ages 40–79 years. The study was carried out in accordance with the Declaration of Helsinki. It was approved by the ethics committee of the University of Leipzig. Participants provided written informed consent prior to participation. Not all subjects participating in the LIFE-Adult-Study performed the entire test battery. In the present study, we analysed the subset of results relating to those participants who completed all chosen neuropsychological tests of the baseline examination 2011–2014. Cognitive assessment, all questionnaires, and OCT measurements have been performed on the same day. We included both eyes per subject wherever possible. Figure 1 illustrates the flow of inclusion/exclusion of participants in the study. We formed two samples. For sample A, which reflects the general population structure, we excluded participants with evidence of eye conditions, either self-reported or inspection of the fundus image. As several neurological or psychiatric conditions can affect brain function, we formed sample B where we additionally excluded the following conditions: relevant tumour, stroke, Parkinson’s disease (PD), multiple sclerosis, epilepsy, depression, and centrally acting drugs.

### Cognitive assessment

Participants were examined with a comprehensive neuropsychological test battery systematically across several cognitive domains (for detailed description, see supplement ‘Methods’ in Girbardt *et al*. 2021;^1^ furthermore, Kynast *et al*. and Luck *et al*.^34-36^). *Subjective memory impairment* was evaluated with a structured computer-assisted interview. We applied the CERAD Plus test battery. The Trail Making Test (TMT) contains two parts: *attention* was assessed with TMT A, requiring participants to draw lines to connect consecutive numbers; *executive function* was assessed using a modification of TMT A, where numbers and letters have to be connected as quickly as possible, related to individual TMT-A performance expressed as a ratio (TMT ratio B/A). *Memory/learning* was assessed with Word List Learning, where participants read 10 printed words and recall as many as possible. *Perceptual-motor abilities* were evaluated by means of the subtest Visuoconstruction Copy, requiring the drawing of figures. *Language* was assessed with the Semantic Verbal Fluency Test, in which participants named as many animals as possible in 1 minute. *Social cognition* was evaluated with the Reading the Mind in the Eyes Test Revised Version (RMET; beyond CERAD Plus), where participants are instructed to identify emotions/mental states by observing the eye region of a face. We applied the Structured Interview for Diagnosis of Dementia of Alzheimer type, Multi-infarct Dementia, and Dementia of other Aetiology according to DSM-III R, DSM-IV, and ICD-10 (SIDAM) to assess an individual’s capacity to perform activities of daily living (SIDAM-ADL scale) and the Center for Epidemiologic Studies Depression Scale (CES-D) to evaluate depression. Education was classified using the Comparative Analysis of Social Mobility in Industrial Nations (CASMIN) scale,^37^ which includes years of education as well as different educational pathways.

### Stratification for mild neurocognitive disorder/mild cognitive impairment

Mild NCD was defined according to the DSM-5 criteria.^17^ MCI was diagnosed according to established consensus criteria.^12,13^ The criteria for both, mild NCD and MCI, are described in detail in Luck *et al*..^36^ In summary, criteria for mild NCD and MCI were (i) evidence of cognitive decline from a previous level of performance as recognized by the participant; (ii) decline in objective cognitive performance, proven by standardized neuropsychological testing with cognitive performance 1–2 standard deviations below age-, sex-, and education-specific norms in at least one of the aforementioned cognitive domains; (iii) preserved independence in everyday activities as operationalized by ≤1 on the SIDAM-ADL scale; and (iv) absence of delirium, major NCD, dementia or another mental disorder (e.g. major depressive disorder, schizophrenia; exclusion criteria operationalized with SIDAM, CES-D, medical history, and cognitive assessment, i.e. for dementia >2 SD deviation from norm in cognitive tests). Age-, sex-, and education-specific norms for the neuropsychological tests were calculated based on the participants’ results in the LIFE-Adult-Study (see Luck *et al*.^38^ and Kynast *et al*.^39^). We furthermore assessed uni-domain (learning/memory) mild neurocognitive disorder and mild cognitive impairment, which we termed amnestic MCI and amnestic mild NCD. Participants analysed here presented solely with loss for the learning/memory domain (=uni-domain). Participants with loss in more than one domain were excluded from these amnestic subanalyses.

Please note that LIFE-Adult participants with major NCD/dementia were neither part of sample A nor sample B, as the focus of the analysis concerned pre-stages of dementia.

Note that in our study, only hippocampal volume was available as a biomarker for AD, but no other more specific biomarkers, such as tau, phosphorylated tau-Tau, and amyloid-β from cerebrospinal fluid or positron emission tomography imaging.

### Ophthalmic assessments

**Figure 2:**
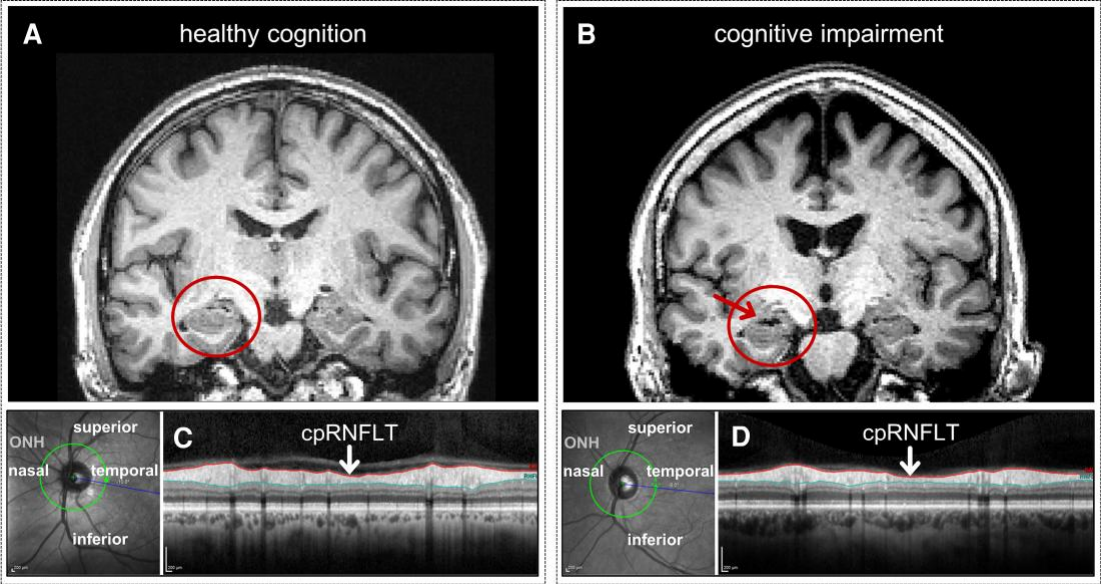
**An example comparison of a participant with normal cognition (A, C) and participant with amnestic mild cognitive impairment (MCI)/amnestic mild neurocognitive disorder (NCD) (B, D).** High-resolution T1-weighted anatomical MR images (**A, B**; voxel resolution = 1 × 1 × 1 mm^3^) showing coronal slices of the brain with a specific focus on the hippocampus region (inset). Note mesial temporal lobe atrophy (arrow) and temporoparietal cortical atrophy of image **B** compared to image **A**. Optical coherence tomography (OCT)-determined circumpapillary retinal nerve fibre layer thickness (cpRNFLT; **C**, **D**). The green circle depicts the location of the measurement around the optic nerve head (ONH) in a fundus image of the left eye. Each clockwise measurement starts temporally. The right OCT-image depicts the B-scan where cpRNFL is located between the red and the blue segmentation lines. Extended information for both OCT-derived images for the participant with normal cognition is presented in Supplementary Fig. 16, and for the participant with amnestic mild cognitive impairment (MCI)/amnestic mild neurocognitive disorder (NCD) is presented in Supplementary Fig. 17. There, the bottom right image illustrates a printout of the Spectralis spectral domain OCT cpRNFLT measurement (black line) depicted on the device-based normative data set (not utilized in this study). Measurements within the green shaded area display the 5th to the 95th percentiles of the device-based normative set (*n* = 201), yellow indicates the 1st to 5th percentile, and red areas depict below the 1st percentile. Note that this section of the machine-printout does not take into account covariates (e.g. age, sex and refraction). Interestingly, in Supplementary Fig. 17, the black line of the amnestic MCI/amnestic mild NCD participant displays much thinner cpRNFLT throughout compared to the cognitive healthy participant in Supplementary Fig. 16. The six sector values (bottom left image in the respective supplement) highlight this. Note: both example participants are part of the examined study samples A and B.

Assessment of ocular health included spectral domain optical coherence tomography (OCT (Spectralis, Heidelberg Engineering, Heidelberg, Germany, Spectralis HRA + OCT, Acquisition Module 5.4.7.0). A circumpapillary OCT was performed to obtain retinal nerve fibre layer thickness with a resolution of 3.9-µm axial and 11-µm lateral, resulting in 768 equidistant measurement points. For more information, see Wang *et al*.^40^. RNFLT is evaluated along the measured circle (see Fig. 2). The high location-specific resolution data were analysed in relation to cognitive assessments. Data analysis of cpRNFLT was carried out only on reliable measurements based on image quality with signal-to-noise ratio ≥20 dB, average number of B-scans ≥50, and no more than 5% missing or unreliable cpRNFLT segmentations among the 768 A-scans. For all OCT scans, unreliable cpRNFLT segmentations were defined as measurements above 99.5% or below 0.5% among the cpRNFLT distribution for all measurement locations. Exclusion criteria were hardware defects, low OCT scanning reliability, and relevant eye pathology, which could have a local impact on RNFLT ^1^. As we previously demonstrated that RNFLT co-varies with age,^40^ with sex,^41^ and with the scanning radius of the measured ring around the optic nerve head^40^ these parameters were integrated in the analysis. The true scanning radius of the measured ring, estimated from the focus of the measurement, is a proxy for ocular magnification. The utilized OCT estimates the true scanning radius^40^ from the individual focus settings of each scan as a measure of ocular magnification.^42^

### Magnetic resonance imaging

MRI of the brain was performed on a 3-Tesla MAGNETOM Verio Scanner (Siemens, Erlangen, Germany). T1-weighted magnetization prepared rapid acquisition gradient echo (MPRAGE), and fluid-attenuated inversion recovery (FLAIR) images were acquired with a standardized protocol: MPRAGE [flip angle (FA) = 9°, relaxation time (TR) = 2300 ms, inversion time (TI) = 900 ms, echo time (TE) = 2.98 ms, 1-mm isotropic resolution, acquisition time (AT) = 5.10 min]; FLAIR TR = 5000 ms, TI = 1800 ms, TE = 395 ms, 1-mm isotropic resolution, AT = 7.02 min]. The following brain parameters were used in this study: left and right hippocampus volume, and white matter lesion volume. Hippocampal volume was based on raw values derived from automated segmentation by the software freesurfer (https://surfer.nmr.mgh.harvard.edu/), which were subsequently adjusted for intracranial volume (ICV). For this, we used the following formula: CVadjusted,i = HCVraw,i−β∗(ICVraw,i−ICVmean) with β as the unstandardized regression coefficient of hippocampal volume (HCV) on intracranial volume (ICV);^43^ see details in Lammer *et al*., 2023.^44^ White matter lesion volume was analysed/acquired by an automated assessment (LesionTOADS) approach, see Lampe *et al*.^45^ for more details.

### Statistical analysis

The cognitive domains were operationalized as *z*-scores for the above-mentioned cognitive tests. Due to the smaller sample size, we analysed the social cognition domain separately.

The associations between RNFLT and the domains of cognitive function were calculated by linear regression with RNFLT as outcome and the cognitive domain score, age, sex, scanning radius and education as regressors. The multivariate regression analyses were carried out with the goal to establish cpRNFLT associations with cognitive performance and especially to find out if cognitive parameters explain variance in addition to those parameters which were previously identified as the strongest impact factors on cpRNFLT (age, sex, scanning radius and education). Our design investigates the necessary conditions to associate cpRNFLT with cognitive decline.

RNFLT was measured separately at 768 equidistant points around the optic nerve head. A *P*-value of less than 0.05 was considered significant after adjustment for multiple comparisons by the false-discovery-rate method.^46^

In addition, we investigated the association between the 768 A-scans of RNFLT and both measures of general cognitive impairment, i.e. MCI and mild NCD. An extracted diagnosis, as described above, of MCI or mild NCD was used as a logistic regression outcome with RNFLT, age, sex, scanning radius and education as regressors; *P*-values were adjusted for multiple comparisons by the false-discovery method. Again, a *P*-value of less than 0.05 was considered significant.

As an additional measure of discriminability, receiver operating characteristic (ROC) curves were calculated for logistic regression models. The *P*-values for the area under the ROC curve (AUC) analyses (Supplementary Fig. 1 and Supplementary Table 4) were calculated by constructing a null model by a permutation test: For each condition, the binary outcome labels were randomly shuffled 5000 times, and for each of these 5000 permutations, our logistic regression models were run and an AUC was calculated. The random permutations of the class labels thereby generate an empirical distribution of ‘null AUCs’, centred around 0.5. We then compared our observed AUC for each respective condition with this distribution of ‘null AUCs’. The *P*-value was calculated as the ratio of the null AUCs greater than or equal to the observed AUC. Supplementary Figs 2–13 show ROC curves for the retinal location with best and worst model performance.

Furthermore, we compared RNFLT between participants with MCI or mild NCD and participants with normal cognition. In the context of this comparison, we also investigated potential differences between MCI and mild NCD in hippocampal volume (known to be related to the most prevalent dementia syndrome, AD)^47^ and white matter lesion load (known to be related to small vessel disease, a further frequent disease associated with aging)^34^ by *t*-tests. These results provide an indication of the likely aetiology of cognitive impairment. We additionally present a separate sex-specific analysis and other subgroup analyses where applicable.

We reanalysed our data following Larrosa and co-workers, who identified optimal cpRNFL sectors to distinguish subjects with AD and healthy subjects with high sensitivity.^48^ To be comparable with their results, we performed our analysis only for sample A, as they did not exclude participants with additional neurologic or psychiatric diseases. In addition, we present our own findings with respect to optimal sectors for our amnestic MCI and amnestic mild NCD cohorts.

## Results

**Figure 3:**
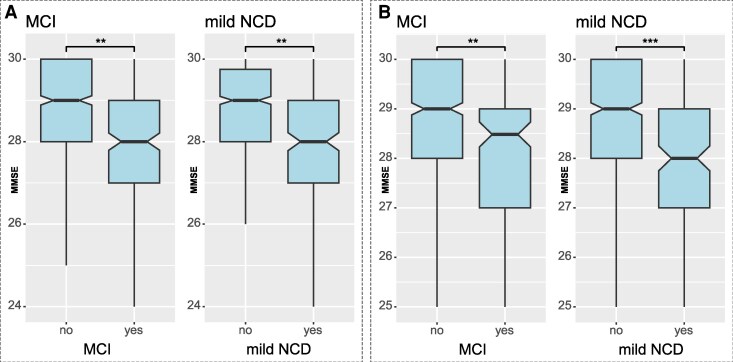
**MMSE Scores presented for participants with normal cognition (no MCI or no mild NCD) and subjects with MCI and mild NCD diagnosis (yes).** MMSE presented with statistically significant differences in participants with cognitive impairment compared to normal cognition. Experimental unit (N) sample A: no MCI = 796, MCI = 229, no mild NCD = 816, mild NCD = 209. Sample B: no MCI = : 658, MCI = 161, no mild NCD = 658, mild NCD = 161, MMSE, mini-mental state examination; MCI, mild cognitive impairment; NCD, neurocognitive disorder, **P < 0,01, ***P < 0001 (*t*-test).

**Figure 4:**
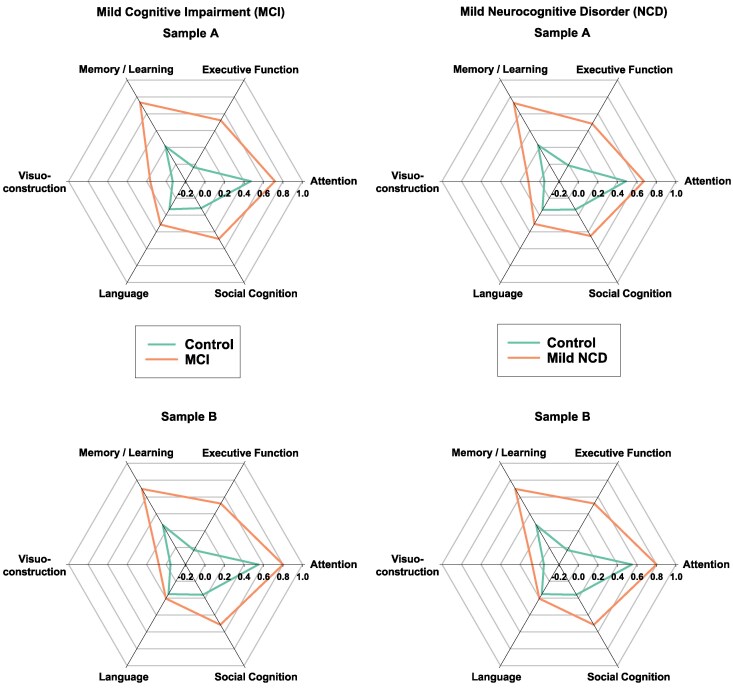
**Impairment in cognitive domains classified as either mild cognitive impairment or mild neurocognitive disorder compared to respective control groups.** Radar plots present mean values after *z*-normalization for the cognitive domains of memory/learning, executive function, attention, social cognition, language and visuoconstruction separately shown for samples A (without eye diseases) and B (without eye disease and additionally without conditions of the central nervous system as listed in Fig. 1). See Table 1 for details on sample size.

**Figure 5:**
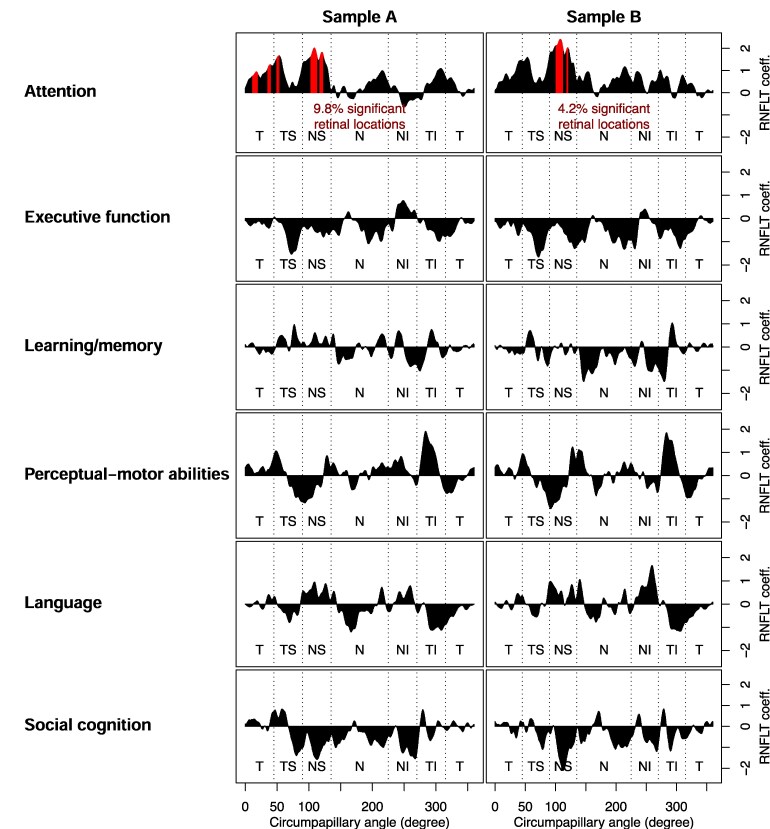
**Association between cognitive performance and circumpapillary retinal nerve fibre layer thickness (cpRNFLT).** Results of domain analysis: Graphical representation of multivariable linear regression analysis for all cognitive domains, adjusted for age, sex, scan radius and education. Association of thicker RNFL with better cognitive performance is depicted with peaks, and association of thinner RNFL with better cognitive performance is depicted with troughs. Pointwise results at 768 angular circumpapillary locations are shown as a joined graph. Sectors are labelled in sequence of measurement with T = temporal, TS = temporal superior, NS = nasal superior, *N* = nasal, NI = nasal inferior, TI = temporal inferior. Statistically significant areas are marked in red. For the domain of attention, 9.8% of the circumpapillary measured areas presented with a thicker RNFL in better results for attention, in sample B 4.2% (note that attention and executive function are presented as TMT-A*(−1) and (TMTB/A)*(−1) to match the graphical representation of the other depicted domains). See Table 1 for details on sample size. The RNFLT coefficients and their corresponding *P*-values (with cognitive domain as outcome) are shown in data Supplementary Table 1. Higher numbers denote thicker cpRNFLT associated with better cognitive performance.

**Figure 6:**
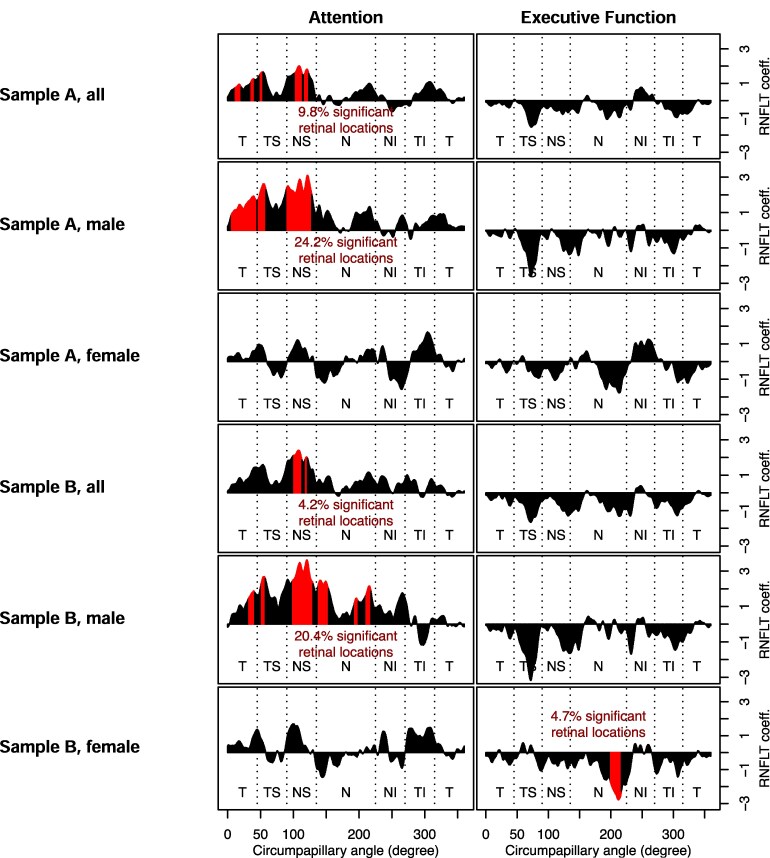
**Association between cognitive performance and circumpapillary retinal nerve fibre layer thickness (cpRNFLT) in attention and executive function domains for female and male subjects.** Graphical representation of multivariable linear regression analysis for all attention and executive function domains, adjusted for age, scan radius and education, depicted for the complete samples A and B, as well as for females and males separately. Thicker RNFLT is depicted as peaks (i.e. thicker cpRNFLT with better cognitive performance for attention), thinner RNFLT with troughs (i.e. thinner cpRNFLT with better cognitive performance for executive function). Pointwise results are shown as a joined graph. Sectors are labelled in sequence of measurement with T = temporal, TS = temporal superior, NS = nasal superior, N = nasal, NI = nasal inferior, and TI = temporal inferior. Statistically significant areas are marked in red. See Table 1 for details on sample size. The RNFLT coefficients and their corresponding *P*-values are shown in data Supplementary Table 2.

**Figure 7:**
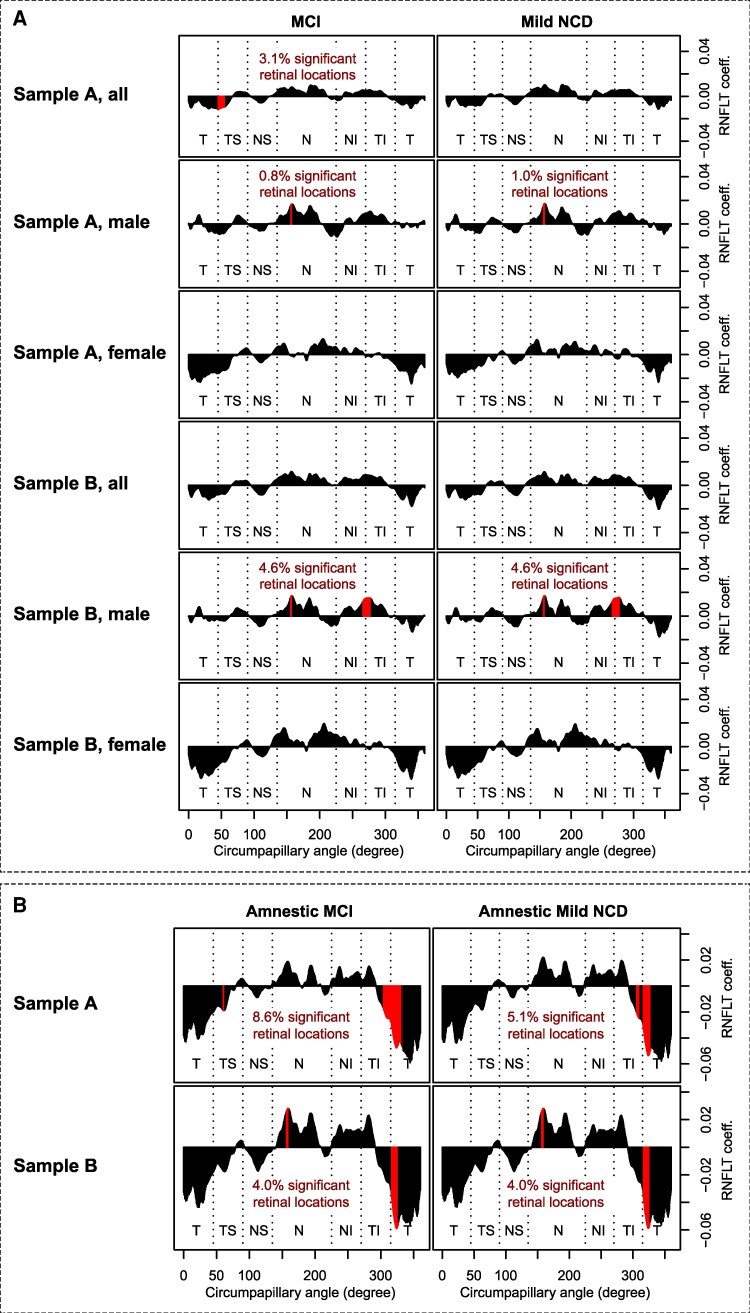
**Association between circumpapillary retinal nerve fibre layer thickness (cpRNFLT) and risk states of dementia, i.e. mild cognitive impairment (MCI) and mild neurocognitive disorder (mild NCD) as outcome.** Graphical representation of logistic regression for MCI and mild NCD (upper panel) and amnestic MCI and amnestic mild NCD (lower panel) adjusted for age, scan radius and education. MCI and mild NCD indicate a cognitive deficit and therefore the outcome is inverted compared to Fig. 6. For the association of MCI or mild NCD with cpRNFLT negative values here means that a thinner cpRNFL is associated with worse cognition, respectively. Thinner cpRNFL in worse cognition is shown as troughs and thicker cpRNFL in worse cognition as peaks. Pointwise results are depicted as a joined graph. Sectors are labelled in sequence of measurement with T = temporal, TS = temporal superior, NS = nasal superior, N = nasal, NI = nasal inferior, TI = temporal inferior. Statistically significant areas are marked in red. See Table 1 for details on sample size. The bottom panel of the Figure depicts uni-domain amnestic MCI: sample A: *n* = 34 (*f* = 19, *m* = 15); sample B: *n* = 25 (*f* = 12, *m* = 13) and uni-domain amnestic mild NCD: sample A: *n* = 30 (*f* = 15, *m* = 15); sample B: *n* = 25 (*f* = 12, *m* = 13), respectively. The axes in both panels of the figure have equal step-size for ease of cross-comparison. The RNFLT coefficients and their corresponding *P*-values are shown in data Supplementary Table 3. For cross-references, raw un-adjusted data are presented in Supplementary Figs 14 and 15.

**Figure 8:**
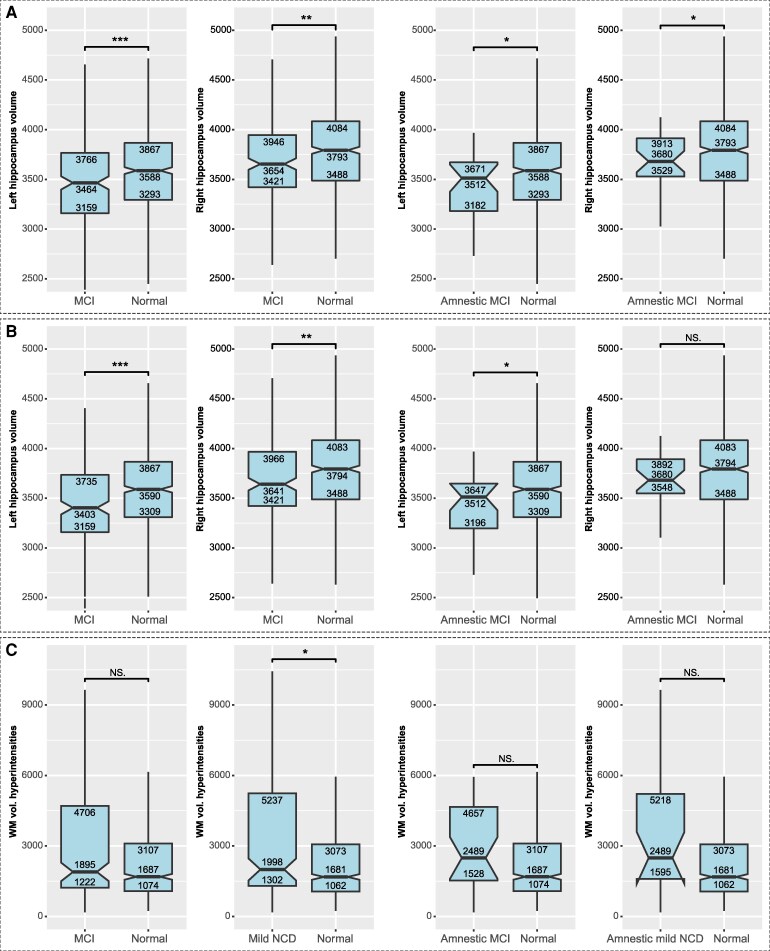
**The results of volumetric studies of the hippocampus and of white matter hyperintensities are shown.** Data from risk states of dementia and from healthy subjects are compared. Boxplots depict the distribution of imaging biomarkers in mild cognitive impairment (MCI), (uni-domain) amnestic MCI [both Figure **A**, MCI *N* = 124, (uni-domain) amnestic MCI *N* = 20], mild neurocognitive disorder (NCD), (uni-domain) amnestic mild NCD [both Figure **B**, mild NCD *N* = 112, (uni-domain) amnestic mild NCD *N* = 18] and healthy subjects. Median and interquartile ranges are shown. **A**: Left hippocampus volume in mm^3^; **B**: Right hippocampus volume in mm^3^, **C**: Volume of white matter (WM) hyperintensities in mm^3^. Experimental unit of **C**: MCI *N* = 116, mild NCD *N* = 104, (uni-domain) amnestic MCI *N* = 19, (uni-domain) amnestic mild NCD *N* = 19] NS non-significant; *<0.05; **<0.01; ***<0.001 (*t*-test).

**Figure 9:**
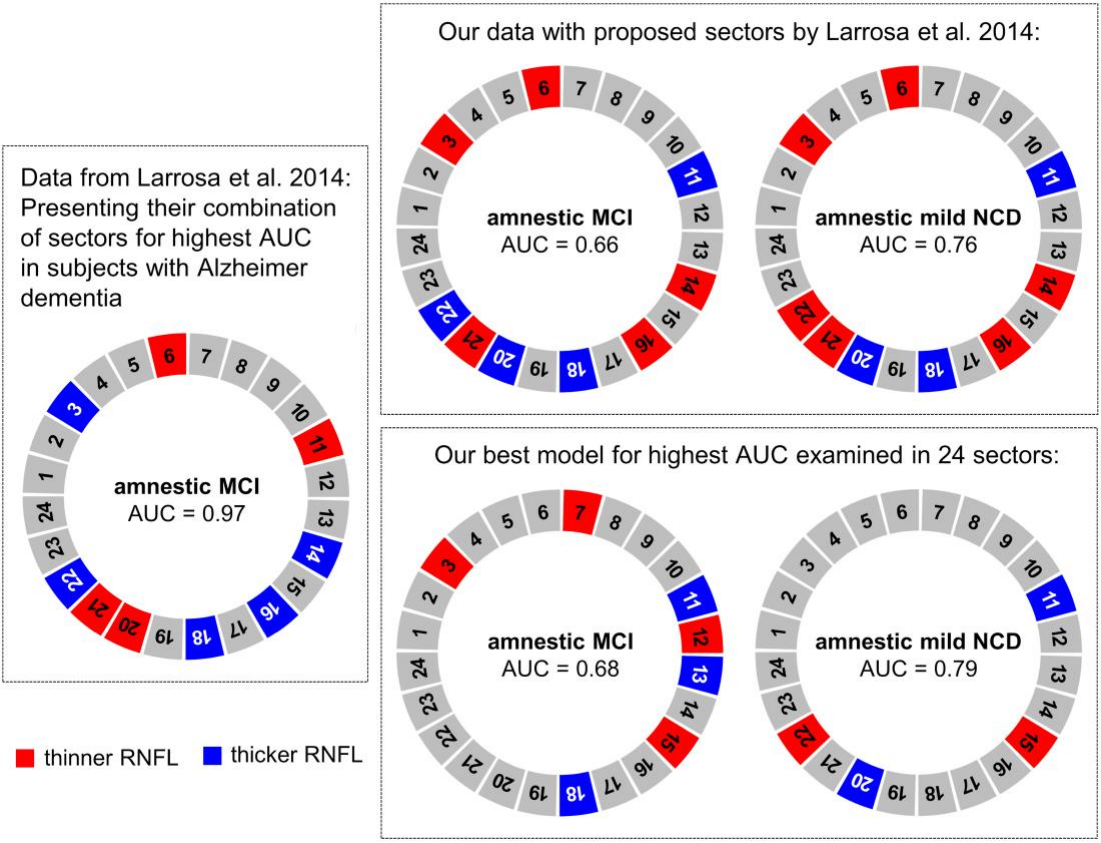
**Graphical representation of data from Larrosa *et al*. (2014), presenting their combination of sectors for the highest area under the curve (AUC) in subjects with Alzheimer’s dementia, in comparison to data from our study following the strategy of their suggested analysis.** Charts depict combination-sector-analysis for predicting Alzheimer’s dementia (Larrosa *et al*. 2014, *N* = 151 eyes of AD patients and 61 eyes of healthy controls) or (uni-domain) amnestic mild cognitive impairment (MCI) and (uni-domain) amnestic mild neurocognitive disorder (NCD) from circumpapillary retinal nerve fibre layer thickness data. Our analysis was performed in sample A to match the exclusion criteria of the Larrosa study. Experimental unit for uni-domain amnestic MCI: *N* = 34 and uni-domain amnestic mild NCD: *N* = 30. This multi-sector approach to establish AUC for a combination of regions is furthermore supported by the RNFLT coefficients (Supplementary Table 1) and their corresponding AUC for our analysis (Supplementary Table 4). See also Supplementary Figs 2–13 for ROC curves for the retinal location with best and worst model performance.

First we introduce demographics of the participants (Samples A and B; Table 1) including mini mental state examination (MMSE, Fig. 3), followed by details concerning cognitive characteristics (Fig. 4). Then, we present the results of the first analyses for RNFLT analysed by cognitive domain for all participants in samples A and B (Fig. 5). Next, the sex-separated analysis in Fig. 6 presents the two domains, where statistically significant differences were found in the prior analysis. This is followed by an analysis for MCI and mild NCD for all participants in samples A and B (Fig. 7). Thereafter, sub-analyses for structural MRI in the same subjects are presented with the aim to elucidate the brain-related origin of cognitive and RNFLT findings. Hippocampus volume and white matter lesions are presented in boxplots analyses (Fig. 8). These important findings are then confirmed by narrowing down the analysis to amnestic MCI and amnestic mild NCD participants in comparison to participants with strictly normal scores (Fig. 7, lower panel). A repetition of the boxplot analyses for hippocampus volume solely in amnestic MCI and amnestic mild NCD participants further underlines this finding (Fig. 8). We present AUC for our analysis in Supplementary Fig. 1, which is followed by a multi-sector approach to establish AUC for a combination of regions (Fig. 9). Supplementary material is available (see Supporting Information file), and specific tables and figures will be called upon in the relevant sections.

**Table 1:** Characteristics of the study population

Sample A	Eyes (% right eye)	Subjects (% male)	Median age in years (2.5%; 97.5%)	Education levels (low; intermediate; high)	MMSE; median (2.5%; 97.5%)	Median global RNFLT (2.5%; 97.5%)
Cognitive Domains: Attention, Executive function, Learning/memory, Perceptual-motor abilities, Language	2133 (50%)	1300 (52%)	69.3 (60.7;78.3)	16%;53%;32%	29 (24.0;30.0)	95 (75.0;113.0)
Cognitive Domain: Social cognition	1288 (50%)	777 (56%)	69.0 (60.6;77.9)	15%;50%;35%	29 (25.0;30.0)	95 (75.4;114.0)
MCI yes	229 (51%)	136 (54%)	70.3 (61.3;78.7)**	16%;47%;37%	28 (23.4;30.0)**	95 (74.4;114.2)
MCI no	796 (51%)	479 (58%)	69.1 (60.7;78.1)**	15%;50%;35%	29 (25.0;30.0)**	95 (76.0;113.0)
mild NCD yes	209 (50%)	123 (58%)	70.3 (61.4;78.8)**	15%;47%;38%	28 (23.0;30.0)*	95 (74.1;113.0)
Mild NCD no	816 (51%)	492 (57%)	69.2 (60.7;78.1)**	16%;50%;35%	29 (25.0;30.0)*	95 (76.3;113.0)

Sample B	Eyes (% right eye)	Subjects (% male)	Median age in years (2.5%;97.5%)	Education levels (low; intermediate; high)	MMSE; median (2.5%; 97.5%)	Median global RNFLT; (2.5%; 97.5%)
Cognitive domains: Attention, Executive function, Learning/memory, Perceptual-motor abilities, Language	1652 (51%)	1003 (55%)	69.1 (60.7;78.3)	14%;52%;34%	29 (25.0;30.0)	95 (75.0;113.0)
Cognitive domain: Social cognition	1005 (51%)	603 (59%)	68.8 (60.6;77.8)	14%;49%;36%	29 (25.0;30.0)	95 (75.0;113.9)
MCI yes	161 (51%)	94 (59%)	70.0 (61.3;78.4)	12%;52%;36%	28 (23.6;30.0)*	96 (74.3;112.3)
MCI no	658 (51%)	395 (59%)	69.0 (60.7; 78.3)	15%;49%;36%	29 (26.0;30.0)*	95 (75.0;113.0)
Mild NCD yes	161 (51%)	94 (59%)	70.0 (61.3;78.4)	13%;52%;35%	28 (23.6;30.0)**	96 (74.3;113.0)
Mild NCD no	658 (51%)	395 (59%)	69.0 (60.7;78.3)	15%;49%;36%	29 (26.0;30.0)**	95 (75.0;113.0)

MCI, mild cognitive impairment; NCD, neurocognitive disorder; MMSE, mini-mental state examination; RNFLT, retinal nerve fibre layer thickness; ∗p<0.05;∗∗p<0.01.

### Demographic characteristics

Table 1 illustrates demographic characteristics of the cohorts. In our first analysis of cognitive domains across all subjects (sample A); we included 2133 eyes of 1300 subjects (mean age: 69.3 years, 95% CI: 60.7–78.3; 52.23% male). For the cognitive domain ‘social cognition’ a subgroup of 1288 eyes of 777 subjects (mean age: 69.0 years; 95% CI: 60.6–77.9; 56.37% male) was examined. For the second analysis of overall cognitive impairment, i.e. with stratification into MCI or mild NCD, we included 1025 eyes of 615 subjects. Table 1 shows detailed information per analysis, and additionally lists the numbers for sample B, where we excluded relevant potentially confounding conditions of the central nervous system (CNS). Generally, the study population consisted almost exclusively of participants of White European descent (>99%).

Education, as classified with the CASMIN-scale,^37^ revealed that 15.69% in sample A and 14.36% in sample B had low education, 52.77% in sample A and 51.74% in sample B had intermediate education, as well as 31.54% in sample A and 33.9% in sample B presented with high education.

MMSE scores shown in Fig. 3 for participants with normal cognition (no MCI or no mild NCD) and subjects with MCI and mild NCD diagnosis, presented with statistically significant differences in MMSE in participants with cognitive impairment compared to normal cognition.

For MCI and mild NCD and their respective control groups (labelled MCI no, mild NCD no), sex and education were equally distributed in both samples (see Table 1). In sample A, participants with MCI (*P* = 0.009) and mild NCD (*P* = 0.004) were older than participants of the respective control groups. There was no age difference in sample B for these groups. In sample A, MCI and mild NCD are present side by side in 120 participants. This means 16 of the 136 participants with MCI did not receive the diagnosis of mild NCD; furthermore, 3 of the 123 participants with mild NCD did not receive the diagnosis of MCI. In sample B, MCI and mild NCD are present side by side in 91 participants. This means 3 of the 94 participants with MCI did not receive the diagnosis of mild NCD; furthermore, 3 of the 94 participants with mild NCD did not receive the diagnosis MCI. Note that all following analyses were adjusted for age, sex, and education and conducted separately for females and males, excluding the impact of these factors on our results.

### Cognitive characteristics

As expected, performance in the MMSE was significantly lower in the MCI (*P* = 0.005 in sample A, *P* = 0.011 in sample B) and mild NCD groups (*P* = 0.012 in sample A and *P* = 0.008 in sample B) than in control subjects, confirming cognitive impairment.

The impact of each cognitive domain on the MCI or mild NCD diagnosis is graphically presented by polar plots in Fig. 4, illustrating cognitive profiles of the participants with either MCI or mild NCD compared to respective control groups. As expected, participants with MCI/mild NCD were impaired in the cognitive domains of attention and memory/learning (in sample A memory/learning was more impaired than attention, in sample B, vice versa). Executive function, social cognition, and language were less affected, with visuoconstruction showing the least impairment.

### Associations between cognitive domains and retinal nerve fibre layer thickness

The first analyses examined the association between performance in the various cognitive domains and cpRNFLT. Results are illustrated in Figs 4 and 5. Note that domains of attention and executive function are presented as TMT-A*(−1) and (TMT B/A)*(−1) to match the graphical representation of the other depicted domains.

As shown in Fig. 5, out of the six cognitive domains, attention, as measured with the TMT-A, correlated significantly with cpRNFLT if the analyses included all subjects. More precisely, a positive association, i.e. thinner cpRNFLT was related to worse cognitive performance, and thicker cpRNFLT was related to better cognitive performance. This association was not only found in sample A, but also in sample B. More precisely, this association of attention with cpRNFLT was detected in 9.8% of measured locations in sample A and 4.2% in sample B when analysing all subjects. Statistically significant effects included segments in the sectors temporal (T), temporal superior (TS) (sample A), and nasal superior (NS) (samples A and B). Analysis of the global mean also led to statistically significant effects (sample A: beta: 0.528, *P* = 0.01; sample B: beta: 0.707, *P* = 0.002). The greatest sectoral changes in descending order for sample A are NS < TS < T. We did not find such associations for the other five remaining cognitive domains. The RNFLT coefficients and their corresponding *P*-values (with cognitive domain as outcome) are shown in data Supplementary Table 1. Higher numbers denote thicker cpRNFLT associated with better cognitive performance.

### Association between cognitive domains and retinal nerve fibre layer thickness in female and male subjects

To take into account possible sex-specific effects, we additionally conducted separate analyses for female and male subjects. Results are shown in Fig. 6 together with the analyses across the whole group, as shown in ST2 for test statistics. Sex-specific associations were detected for attention and executive function, without significant findings for memory/learning, perceptual-motor abilities, language, and social cognition (latter domains, accordingly, not shown in the Figure).

The association between the domain attention and cpRNFLT (Fig. 6, left column) was driven by the male group, where 24.2% of retinal locations in sample A and 20.4% in sample B showed a significant correlation of thinner cpRNFL with worse cognitive performance and vice versa, thicker cpRNFL with better cognitive performance. The NS segment showed associations in the majority of the measurement points for males in both samples. Whereas in sample A additionally, sector T showed broad effects, in sample B, only a narrower section was related to cognition. No such association was detected for the female group (Fig. 6, right column). Note that the result for attention and cpRNFLT in male subjects obtained in sample A was also found in sample B.

Results for the association between the domain executive function and cpRNFLT were not statistically significant, except for a statistically significant association of worse cognitive performance with thicker cpRNFL in 4.7% circumpapillary locations in females of sample B (see Fig. 6, right column). These sections are all located in the nasal peripapillary sector. In male subjects and the analyses across the whole cohort, this link was not present.

Note, if one visually inspects the troughs and peaks of attention and executive function in Fig. 6, they occasionally look reciprocal (with local maxima of attention corresponding to local minima of executive function). The potential reason is that we observed a negative correlation between attention and executive function, i.e. performance in TMT-A and TMT-B/A with −0.2109, *P* < 0.001. When adjusted for age, this association was slightly stronger with −0.2188; *P* < 0.001. This finding mirrors the visually observed reciprocal association between attention/executive function and cpRNFLT.

### Association between retinal nerve fibre layer thickness and risk states of dementia

Next, we investigated the impact of risk-states for dementia, i.e. mild NCD and MCI, on cpRNFLT. Results are illustrated in Fig. 7, as shown in ST3 for test statistics. MCI and mild NCD indicate a cognitive deficit and therefore the outcome is inverted compared to Fig. 6. Thinner cpRNFL was linked to MCI in 3.1% retinal locations of sample A, where thinner cpRNFL predicted MCI. When analysing all subjects of sample A, these locations were found in the temporal-superior (TS) sector. Sub-analyses with stratification for sex showed additional but small effects in men, however, with a different direction of the effect. 0.8% locations in sample A and 4.6% locations in sample B presented with thicker RNFL nasally (*N*) (samples A and B) and inferiorly (segments nasal inferior/temporal inferior, NI/TI) (sample B) for subjects with MCI.

Compared to MCI, the results for mild NCD presented an overall similar profile of associations. However, no statistical significance was observed when analysing the whole samples A and B across female and male subjects. Sub-analyses for sex presented again an effect for male subjects; 1.0% locations in sample A and 4.6% locations in sample B presented with thicker cpRNFL nasally [within sector N (samples A and B)] and inferior [within sectors nasal inferior/temporal inferior, NI/TI (sample B)] for subjects with MCI. Female subjects overall did not show associations between cpRNFLT and risk states of dementia, i.e. neither for MCI nor for mild NCD.

It is well known that AD represents the most frequent aetiology for cognitive dysfunction and dementia in aging.^47^ Therefore, we compared RNFLT between participants with amnestic MCI or amnestic mild NCD—the risk state for Alzheimer's dementia^12^—and participants with normal cognition in a logistic regression with two groups. In contrast to Fig. 5, where we associate learning/memory versus RNFLT regardless of possible deficits, the analysis of Fig. 7 revealed that amnestic MCI and amnestic mild NCD were mainly associated with thinner RNFLT. In detail, thinning was found in the temporal and temporal-inferior regions. In sample B, we additionally found a small peak nasally. Sex-specific analyses were not applicable due to the low numbers of participants.

When comparing the upper and lower panels of Fig. 7, it can be seen that the association between cpRNFLT and MCI or mild NCD, respectively, is stronger in participants with amnestic MCI or amnestic mild NCD presented in the lower panel. Thicker RNFL is depicted with peaks, thinner RNFL with troughs. For MCI and mild NCD, troughs are present in the temporal regions, specifically for female participants. This effect becomes much more pronounced and statistically significant when analysing data for amnestic MCI or amnestic mild NCD, as shown in lower panel of Fig. 7.

### Association between risk states of dementia and brain imaging biomarkers

In addition to AD, small vessel disease is a further common disorder associated with aging, which is evidenced by white matter lesions on structural MRI.^34^ To elucidate the aetiology of MCI and mild NCD in our study cohort, we compared white matter lesion load and hippocampal volume in participants with these cognitive states with healthy subjects, as indicators or biomarkers of either underlying small vessel disease^34^ or AD.^47^ Results are illustrated in Fig. 8. Notably, this analysis revealed statistically smaller hippocampal volumes in both MCI and mild NCD, compared to cognitively normal participants. This was mainly absent for white matter lesion load, indicating evidence for predominantly AD aetiology. The same findings were obtained for amnestic MCI and amnestic mild NCD.

### Area under the curve in receiver operating characteristic analysis

Next, we investigated how well cpRNFLT predicts amnestic MCI and amnestic mild NCD, respectively. Covariates were accounted for as described in the methods section. Detailed results are shown in Supplementary Table 4 and Supplementary Fig. 1 (see also Supplementary Figs 2–13 presenting ROC curves for the retinal location with best and worst model performance).

#### Pointwise location analysis

Data were analysed from all 768 pointwise circumpapillary locations. AUC for ROC analysis was found to be location specific. Substantial differences existed across 768 examined locations. The AUC ranged from 0.62 (minimum across all locations) to 0.81 (maximum) depending on location, sample or sex. The maxima for AUC on cpRNFLT predicting amnestic MCI or amnestic mild NCD ranged between 0.73 and 0.81 (all are associated with locations in the temporal or temporal-superior sectors in female participants). For the same analysis settings, the lowest predictive regions (AUC minima) corresponded to nasal locations. Additionally, a superior region presented with low predictive power when results from males and females were combined (in sample A for both amnestic MCI and amnestic mild NCD).

#### Sector-specific analysis

In the sector-specific analysis, AUC maxima for cpRNFLT predicting amnestic MCI or amnestic mild NCD were found in the temporal sector (T) for all investigated groups (maximum AUC ranged between 0.69 and 0.80), indicating that this sector shows the highest predictive value. For the total sample A with cpRNFLT predicting amnestic MCI, almost no sector-specific location differences were observed (AUC 0.64–0.70 for global and individual sectors). The highest predictive values were found in the temporal sector (T). For the total sample B, sector-specific differences ranged between AUC 0.65 and 0.72, with the most predictive location being again the temporal sector (T). Comparable results were observed for amnestic mild NCD (AUC 0.62–0.71 for sample A and AUC 0.65–0.72 for sample B).

### Area under the curve in receiver operating characteristic analysis based on a combination of sectors

Larrosa *et al*.^48^ presented interestingly high AUC values for the detection of Alzheimer’s disease promising approach with a combination of 24 cpRNFLT sectors. As proposed by them, we investigated how well cpRNFLT predicts amnestic MCI and amnestic mild NCD, respectively, when investigating this relationship for a group of cpRNFLT sectors combined (Fig. 9). This analysis was performed in sample A. In the aforementioned study, the best AUC to predict Alzheimer's dementia was reached with a specific combination of nine out of 24 circumpapillary sectors.

First, we present our results when using the same nine sectors proposed by Larrosa and co-workers. AUC for amnestic MCI is in this case 0.66, and for amnestic mild NCD AUC, it is 0.76. Second, we present the best model of combined cpRNFLT sectors for our data. cpRNFLT predicts amnestic MCI with a combination of seven segments reaching an AUC of 0.69. Akaike's information criterion (AIC) is 515.24 and Bayes information criterion (BIC) is 553.46. For amnestic mild NCD, the highest AUC is reached with a combination of only four segments. AUC is 0.79, AIC is 245.06, and BIC is 268.77. In the analysis of Larrosa *et al*. as well as in our four analyses, both thinner and thicker RNFLT in different segments are present. Both analyses were carried out adjusting for co-variables as described in the methods. As Larrosa *et al*.^48^ also present the raw un-adjusted data in their manuscript; thus, we include Supplementary Figs 14 and 15 to depict our results in their format as well.

## Discussion

Our study investigated the association between cpRNFLT and cognition in a large population-based dataset, including more than 1000 subjects with an age range from 60 to 78 years of age. Remarkably, we systematically included all cognitive key domains of DSM-V, ranging from attention, memory/learning, executive function, visuoconstruction, and language to social cognition, to enable deep-phenotyping. In contrast, former studies mainly focused on single or a few domains, i.e. the memory/learning domain associated with Alzheimer’s dementia.^47^ Of note, the language and, in particular, social cognition domains are generally under-investigated, presumably related to an Alzheimer-centred focus in former neurodegeneration research.^49,50^ Social cognition has been taken into consideration in research in recent years, where its relevance increased not only in general aging but also in age-related diseases such as small vessel disease or early-onset neurodegenerative diseases (e.g. frontotemporal dementia).^34,35,51^ Moreover, we investigated the association between cognition and cpRNFLT in great detail beyond approaches limited to sectors, as done in former studies.^52^ Although of high relevance to the underlying anatomy, there is no *a prior* reason to suppose that the sectors as defined will inevitably show disparate involvement in any particular disease process. As our results show, there may be advantages in a more fine-grained analysis.

### Thinner retinal nerve fibre layer is associated with worse attention, driven by male subjects

Our results show that cpRNFLT is associated with the cognitive domain of attention as measured with the TMT-A. Here, a thinner cpRNFL was related to worse performance in attention, and a thicker cpRNFL was associated with better performance in attention. This finding was confirmed in sample B, where we excluded participants with brain-relevant diseases and/or exposure to psychoactive drugs. The finding encompassed the temporal, temporal-superior and nasal-superior sectors. In addition to sector-specific, within-sector findings, this effect was confirmed for the global mean of cpRNFLT. We did not find positive associations for the other five remaining cognitive domains. For the sole domain of executive function, we observed some negative correlations. It is unclear if those are inconsistent findings or represent different affecting biological/neurological mechanisms (see discussion below). The aforementioned relationship between cpRNFLT and attention was ascribed to male subjects in the sex-specific analyses, without effects in female subjects. In sum, our results show that cpRNFLT is associated with attention and executive function in a sex-specific manner and that this parameter may predict risk states of dementia. Findings are very promising but warrant further evidence for valid prediction of dementia risk states from cpRNFLT. Existing differences in prevalence of the various causes of dementia between women and men (e.g. AD is more common in women, PD and vascular dementia are more common in men) may indicate that in this cross section of younger asymptomatic individuals, the findings would differ in males and females. With respect to previous findings, we note that one Chinese study involving 96 patients with AD also revealed an association between attenuated global RNFLT and worse attention (also measured by TMT-A as in our study). Additionally, these subjects with abnormal RNFLT scored lower in mini-mental state examination (MMSE), memory, language, and executive function, as expected. However, the study did not include a healthy control group, and no sector-wise analysis was conducted.^53^

Other studies examined these associations in healthy cohorts. Mammadova *et al*. investigated RNFLT and cognitive domains in a small sample of 20 healthy older subjects. Here, RNFLT in the temporal quadrant was positively correlated with working memory and selective attention, which were measured with the Wechsler Adult Intelligence Scale (WAIS) Digit Span Backward test.^26^ Furthermore, another study established positive correlations between almost all the examined cognitive domains and retinal parameters.^54^ These data were obtained by scanning laser polarimetry. In contrast, neither an analysis in 227 non-demented elderly participants in an ongoing Taiwanese cohort study^55^ nor a limited evaluation of nine participants with AD and nine with MCI^56^ found an association of global RNFLT with domain-specific cognition. Regarding our findings within temporal, temporal-superior and nasal-superior sectors, a focus of thinner superior and inferior RNFL in patients with cognitive deterioration has been found in several studies, see meta-analysis,^57^ but these did not evaluate correlation with specific domains.

Other than the impact of attention on RNFLT, only one further statistically significant result was obtained in our sex-specific analyses, here an association of thicker cpRNFLT within the nasal sector with worse cognitive performance for executive functions in females after controlling for the impact of brain-relevant diseases and psychoactive drugs. However, this sex-specific finding was not confirmed in the whole sample, and all remaining analyses included both females and males. Moreover, executive functions were operationalized as the TMT-B/TMT-A ratio, where we found a negative correlation between attention and executive function, i.e. performance in TMT-A and TMT B/A. Accordingly, the association between executive function and cpRNFLT in our study might, at least in part, be an effect of the relationship between attention and executive function. On the other hand, associations of worse cognition with locally thicker RNFLT have also been described by others.^19^ It is not clear if these negative correlations are the result of RNFL thickening over time in subjects with deficits in executive function or reflect static anatomical differences between the groups. Furthermore, in the aforementioned case–control study of nine patients, respectively, with AD and MCI with a one-year follow-up, besides a significant linear trend of thinning in superior and temporal RNFLT, a thickening has been found in the nasal segment of subjects with progressive cognitive decline.^56^ Of note, the above-mentioned studies had a much smaller sample size as well as much lower resolution in RNFLT analysis than our study and did not deep-phenotype their cohorts such as in our study.

Of note, approximately 10% of our participants showed cognitive impairment, i.e. mild NCD or MCI, as risk states for dementia.^47^ The profile revealed impairment principally in the cognitive domains of attention and memory/learning in our group of mild NCD and MCI, followed by executive function, social cognition and language. Visuoconstruction was the domain with the lowest impairment. Attention, therefore, was one of the most impaired cognitive domains, which might have supported the stronger correlation between cpRNFLT and this domain due to higher statistical power.

In summary, our large study demonstrates that thinner cpRNFLT is consistently associated with worse performance in the cognitive domain of attention. This finding is mainly driven by the male subset of subjects. This was obtained in samples A and B. We did not find such associations for the other five remaining cognitive domains. A trend was observed for women in sample B for an association between worse cognitive performance and thicker cpRNFL for executive functions. The validity of our findings is underlined by the large cohort, cognitive deep-phenotyping, and the high spatial resolution of cpRNFLT.

### The association of retinal nerve fibre layer thickness with cognitive performance showed no specific laterality effects of left and right eyes

We included both eyes per subject wherever possible. Left and right eyes were balanced, as given in Table 1. We have previously described in the same dataset in a high level of detail the similarities and differences between right and left eyes within the same person.^58^ Therefore, we performed separate data analyses for left and right eyes but did not find significant differences with respect to the outcomes investigated in this study.

### Retinal nerve fibre layer thickness is associated with risk states of dementia, i.e. mild cognitive impairment and mild neurocognitive disorder

Beyond the analysis of the association between cpRNFLT and the several cognitive domains, we investigated the question: does cpRNFLT predict risk states of dementia, i.e. MCI and recently introduced category of mild NCD.^47^ Including both dementia-risk states in our study enabled comprehensive embedding of results in the available literature. First investigations for MCI and mild NCD (upper panel Fig. 7) revealed that thinner cpRNFLT in the temporal-superior segment is linked to MCI for sample A. This finding corresponds to the results from the first analysis, because the association between thinner cpRNFLT and impaired attention included this segment. This finding was not present in sample B, possibly due to the smaller sample size (reduction by around 25%).

Specific analyses for amnestic MCI and amnestic mild NCD (lower panel in Fig. 7) revealed that thinner cpRNFLT within temporal sectors is linked to amnestic MCI for sample A; likewise, cpRNFLT predicted amnestic mild NCD within the same sectors. This finding was confirmed in our sample B with the exclusion of participants with brain-relevant diseases and/or receiving psychoactive drugs. This finding corresponds to the results from the first analysis (Fig. 5), because the association between thinner cpRNFLT and impaired attention included this segment. Furthermore, a nasal thickening was observed for amnestic mild NCD in sample B. Moreover, the ROC analysis (Supplementary Tables 1 and 4) revealed that cpRNFLT could predict amnestic MCI or amnestic mild NCD (ranging from 0.72 to 0.81 for AUC), corresponding to the temporal (and temporal-inferior) region.

Several smaller cross-sectional studies found thinner RNFL in primarily superior and inferior quadrants in subjects with MCI compared to healthy controls, as shown in a recent meta-analysis.^57^ However, these studies did not analyse RNFL in higher resolution and did not cover all cognitive domains systematically. To our knowledge, the more recent classification of mild NCD with in-depth phenotyping according to DSM-5 has not yet been investigated with respect to RNFL.

Regarding sex-specific analyses (upper panel Fig. 7), we detected effects for the male but not the female sample, mirroring the male-dominant results in the association analyses between cpRNFLT and attention (see first subsection of the Discussion). Of note, effects were consistent for MCI and mild NCD here. In comparison to the sample A analysis for MCI, effects showed another direction and were located in other segments. In detail, MCI and mild NCD were related to thicker cpRNFLT within nasal, nasal inferior and temporal-inferior sectors in male subjects. One might assume a correspondence to the association analyses as discussed above. Although this analysis detected an association of thicker cpRNFLT with worse cognitive performance for executive functions, this finding was limited to female subjects and located in the nasal sector, but in a different part of this segment. In Fig. 7, lower panel, the comparatively smaller sample size reduces the power of our statistical data analyses, compared to most other analyses shown in this paper. It is noteworthy that the effect mostly survives even for the smaller sample B (*n* = 25) and, notably, at the same locations as in the larger sample A (*n* = 34). Given these results, it is possible that the size of our effects is underestimated, and the effects would even be stronger with larger sample sizes. Regarding ROC analyses (Supplementary Tables 1 and 4), the temporal (and temporal-inferior) sector reached maxima AUC for cpRNFLT to predict mild NCD or MCI for sample A and sample B, when separated by male and female groups, which corresponds to these findings. However, generally, we expected thinner RNFL to be associated with risk states for dementia, i.e. MCI and mild NCD. Accordingly, the partly inverse associations as detected in our study require further studies confirming these findings and elucidating associated mechanisms.

Most former published studies adjusted results for sex or presented sex-specific differences in RNFLT. But to our knowledge, no studies presented the correlation of cognition and RNFLT for females and males separately—an issue discussed as important by Li and Rauscher *et al*. 2020.^41^ Several studies found thinner RNFLT in patients with AD compared to healthy controls.^59,60^ Additionally, longitudinal studies gave evidence of a reduction in RNFLT over time with a decline in the memory domain.^61^ As AD is the most prevalent type of dementia, we had expected to see an effect in the memory domain. The lack of associations for memory in the regression analyses might be related to the lack of power due to our strict exclusion of dementia or major NCD cases in our study.

Histologically, it has been observed that RNFL, as well as other retinal layers, decline in patients with AD.^62,63^ Asanad *et al*. found significant thinning superiorly (temporo-superior and nasal-superior) for all observed layers.^62^ Deposits of amyloid beta plaques, typically associated with AD, have fittingly also been described in the superior segment.^8,9^ Hyperphosphorylated tau has also been detected in the retina of patients with AD.^64,65^ Neuroanatomically, fibres from the superior retina project to the cuneal gyrus of the primary visual cortex, whereas fibres from the inferior retina project to the lingual gyrus. Armstrong *et al*. found a greater density of senile plaques and neurofibrillary tangles in the cuneal gyrus than in the lingual gyrus in AD, which corresponds to the superior retina and fits well with our results.^66^ This phenomenon might also explain the previously described inferior visual field defects in AD.^67^ Regarding our result of thicker cpRNFLT in MCI and mild NCD in male subjects, studies are of interest showing an augmentation of this retinal layer in MCI compared to healthy adults.^19,68^ The authors hypothesized that this effect might be related to gliosis preceding neuron loss/axonal degeneration and tissue thinning.

Notably, we used brain imaging biomarkers to relate our results to the potential aetiologies of mild NCD or MCI. The more common causes for cognitive decline generally are age-related diseases such as incident AD with mainly mnestic, but also executive and attentional deficits,^12,47,69^ and small vessel disease with deficits in attention, memory/learning and social cognition.^34^ Less likely are various rare neurodegenerative diseases, metabolic diseases, and psychiatric disorders with widespread and less distinct cognitive impairment. Participants with mild NCD and MCI in our study showed mainly impairments in the cognitive domains of attention and memory/learning. Our in-depth brain imaging biomarker analyses revealed that hippocampal volume as an indicator for AD^47^ was reduced in MCI and mild NCD, but to a much lesser extent than white matter lesion load as an indicator of small vessel disease.^34^ These results support the assumption that—together with the high incidence of such diseases in the aging population—aetiology in our cohort was mainly related to AD and to a much smaller extent to small vessel disease. This hypothesis is further supported by the fact that we excluded as far as possible confounding diseases affecting the brain/mind and its cognition: conditions such as tumour, stroke, Parkinson's disease, multiple sclerosis, depression and consumption of centrally acting drugs were excluded in the maximally controlled sample B. It would be great to correlate our findings on the RNFLT regionally specific with brain imaging data, such as structural magnetic resonance imaging (sMRI) combined with voxel-based morphometry (VBM). Here, we would expect specific relationships. However, we had only limited volumetric information for specific brain regions.

### Retinal nerve fibre layer thickness might predict risk states of dementia, i.e. mild cognitive impairment and mild neurocognitive disorder—but future studies are warranted

Finally, we want to discuss the results of the ROC analyses, namely the question of whether cpRNFLT might predict risk states of dementia, i.e. MCI and mild NCD. ROC analyses were conducted across the 768 pointwise locations and for the sectors. For the pointwise-specific analyses, AUCs (stratified for sex) were location-specific and reached maxima between 0.63 and 0.65 for mild NCD, with minimum values of 0.56. Slightly smaller but very comparable results were observed for MCI. For the sector-specific analyses, AUCs reached maxima from 0.62 to 0.63 for mild NCD, again with slightly smaller but very comparable results for MCI. Sectorial AUC analyses gave the strongest results for temporal and temporal-inferior location, coinciding with the pointwise analysis, which identified the second cpRNFLT peak as being most predictive.

A recent study applying comparable approaches (OCT and RNFLT) reached higher AUC values in separation between AD and healthy controls, when combining RNFLT and ganglion cell layer (AUC 0.874).^70^ Remarkably, Larrosa *et al*. stepwise grouped several circumpapillary locations and consequently reached a maximal ROC of 0.97 to distinguish between AD and healthy individuals.^48^ For their study, this resulted in nine most promising out of 24 designated sectors, each 15° wide, which included thinner and thicker RNFLT regions when comparing patients with Alzheimer’s dementia with controls. We repeated their pattern analysis on our data, using their proposed sectors as well as creating new sectors from our raw data. However, when examining their nine specific sectors for our data, the AUC remained low. The likely reason for these differences is the fact that Larrosa and co-workers examined patients with Alzheimer’s dementia in a case–control study, whereas our analysis was based on a population sample which included patients with (amnestic) MCI and/or with mild (amnestic) NCD. A lower incidence of true positives is to be expected in pre-morbid than in established AD.

When establishing our best model by selecting from the 24 sectors for our study sample, we obtained higher AUCs. This is especially promising for amnestic mild NCD, where a combination of only four segments predicted impaired cognition from cpRNFLT. Differences between amnestic mild NCD and amnestic MCI might be related at least in part due to non-overlapping groups of subjects. Interestingly, several findings of Larrosa *et al*. were overlapping but inverted (opposite mathematical directions) to our results, which might stem from differences in controlling for confounding factors, e.g. adjustment for refractive error which can result in circumpapillary shift/displaced cpRNFLT peaks in myope or hyperope participants. Nevertheless, these alterations are localized in similar segments to those proposed by Larrosa *et al*.^48^ This demonstrates that they identified vulnerable spots of the RNFLT to distinguish between Alzheimer’s dementia and healthy subjects. Astonishingly, in some segments, our signs are inverted compared to Larrosa *et al*.^48^ A reason therefore might be the lack of adjustment of confounders by Larrosa *et al*. or might be caused by stepwise dynamic development of the disease from pre-stages to dementia.

Note that in both studies, the number of subjects was limited; thus, the findings will benefit from validation in larger studies. One further aspect to consider is the absence of longitudinal data to examine whether subjects convert to Alzheimer's dementia.

To our knowledge, only one study calculated AUC including patients with MCI. Using RNFLT alone, AUC was 0.68 to separate between MCI and healthy control, increasing to 0.79 when adding macular layers and multiple ocular factors.^71^ This supports the relevance of combining inner retinal layers as in the above-mentioned study.^70^

## Conclusion

Our results show that cpRNFLT is associated with attention and executive function in a sex-specific manner, revealing the more vulnerable regions related to CNS pathology, and that this parameter may predict risk states of dementia. This results in OCT being a non-invasive and rapid objective method valuable for population screening and monitoring of treatment. Findings are very promising but warrant further validation. In future studies, longitudinal data have to be taken into consideration as only a minority of participants with MCI or mild NCD later develop dementia or major NCD (so-called converters),^47,72^ whereas the majority remains stable or even improves after sufficient treatment of, for instance, metabolic or psychiatric disorders (so-called non-converters).^47,72^ Accordingly, future specific analyses need to be conducted after prospective stratification into these groups. This procedure based on longitudinal studies might lead to developing cpRNFLT as a tool to predict cognitive decline in patients. A further refinement, going beyond our approach (conducting ROC analyses for single points or sectors), the application of machine learning and artificial intelligence such as support vector machines or deep neural networks has the potential to raise statistical power and, hence, sensitivity.

## Supplementary Material

fcaf464_Supplementary_Data

## Data Availability

Raw data cannot be shared publicly because of consent restrictions of LIFE-Adult-Study participants. Data are available after an approved project agreement from the LIFE Leipzig Research Center for Civilization Diseases. Please file a proposal with info-life@lists.uni-leipzig.de for data access requests.
