# Supplementary material for: Retinal nerve fibre layer thickness is associated with attention and predicts risk states of dementia
Source: Brain Commun. 2025 Dec 5;7(6):fcaf464. doi: 10.1093/braincomms/fcaf464 (PMC12679710; doi:10.1093/braincomms/fcaf464)
Supplement: fcaf464_Supplementary_Data [file fcaf464_supplementary_data.zip › Supplementary Materials.pdf]

Supporting Information to

Retinal nerve fibre layer thickness is associated with attention and predicts risk states of dementia

Short running title: Retinal layer and dementia risk states

Matthias L. Schroeter, MD[1,2,3*], Johanna Girbardt, MD[1,4*], Tobias Luck, PhD[5], Francisca S. Rodriguez, PhD[6,7], Gordon T. Plant, MD[8], Barbara Wicklein, Dipl.-Ing.[4], Kerstin Wirkner, PhD[3,4], Christoph Engel, MD[3,4], Jana Kynast, PhD[1], Christian Girbardt, MD[9], Mengyu Wang, PhD[10], Maryna Polyakova, PhD[1], Andreas Hinz[11], A. Veronica Witte, PhD[1,3], Toralf Kirsten, PhD[12,3,4], Markus Loeffler, MD[3,4], Arno Villringer, MD[1,2,3], Steffi G. Riedel-Heller, MD[6,3], Tobias Elze, PhD[10,3,#], Franziska G. Rauscher, PhD[3,4,12;§,#]

[1] Department of Neurology, Max Planck Institute for Human Cognitive and Brain Sciences, Leipzig, Germany
[2] Clinic of Cognitive Neurology, Leipzig University Hospital, Leipzig, Germany
[3] Leipzig Research Centre for Civilization Diseases (LIFE), Leipzig University, Leipzig, Germany
[4] Institute for Medical Informatics, Statistics and Epidemiology, Leipzig University, Leipzig, Germany
[5] Faculty of Applied Social Sciences, University of Applied Sciences Erfurt, Erfurt, Germany
[6] Institute of Social Medicine, Occupational Health and Public Health (ISAP), Leipzig University, Leipzig, Germany
[7] German Centre for Neurodegenerative Diseases (DZNE), Research Group Psychosocial Epidemiology and Public Health, Greifswald, Germany
[8] Institute of Neurology, University College London, London, UK
[9] Department of Ophthalmology, Leipzig University Medical Center, Leipzig, Germany
[10] Schepens Eye Research Institute, Harvard Medical School, Boston, MA, USA
[11] Department for Medical Psychology and Sociology, Leipzig University Medical Center, Leipzig, Germany
[12] Medical Informatics Center - Department of Medical Data Science, Leipzig University Medical Center, Leipzig, Germany

* Matthias L. Schroeter and Johanna Girbardt, MD contributed equally
# Tobias Elze and Franziska G. Rauscher contributed equally

Corresponding author:
Dr. Franziska Rauscher
Leipzig University
Institute for Medical Informatics, Statistics and Epidemiology
Härtelstraße 16–18
04107 Leipzig
Germany
E-Mail: franziska.rauscher@medizin.uni-leipzig.de

Supplementary Table 1: **Association between cognitive performance and circumpapillary retinal nerve fibre layer thickness (cpRNFLT)**

See excel spreadsheet Supplementary Table 1 tab A (sample A) and tab B (sample B) for cpRNFLT coefficients and p-values per angular location (corresponding to Figure 5).

Supplementary Table 1: Association between cognitive performance and circumpapillary retinal nerve fibre layer thickness (cpRNFLT). The RNFLT coefficients (with cognitive domain as outcome) are shown in Supplementary Table 1 (p-values from column ACP). Greater numbers denote thicker RNFL associated with better cognitive performance. The RNFLT coefficients (Supplementary Table 1) and their corresponding AUC for our analysis (Supplementary Table 4) are followed by a multi-sector approach to establish AUC for a combination of regions (Figure 9).

Supplementary Table 2: **Association between cognitive performance and circumpapillary retinal nerve fibre layer thickness (cpRNFLT) in attention and executive function domains for female and male subjects**

See excel spreadsheet Supplementary Table 2 for cpRNFLT coefficients and p-values per angular location (corresponding to Figure 6).

Supplementary Table 2: Association between cognitive performance and circumpapillary retinal nerve fibre layer thickness (cpRNFLT) for female and male subjects. The RNFLT coefficients (with cognitive domain as outcome) are shown in Supplementary 2 (p-values from column ACP). Greater numbers denote thicker RNFL associated with better cognitive performance.

Supplementary Table 3: **Association between circumpapillary retinal nerve fibre layer thickness (cpRNFLT) and risk states of dementia, i.e. mild cognitive impairment (MCI) and mild neurocognitive disorder (mild NCD) as outcome**

See excel spreadsheet Supplementary Table 3 for cpRNFLT coefficients and p-values per angular location (corresponding to Figure 7).

Supplementary Table 3: Test statistics for logistic regression for MCI and mild NCD as well as (uni-domain) amnestic MCI and (uni-domain) amnestic mild NCD adjusted for age, scan radius and education. The RNFLT coefficients are shown (p-values from column ACP).

Supplementary Table 4: **Circumpapillary retinal nerve fibre layer thickness (cpRNFLT) predicts amnestic MCI and amnestic mild NCD**

See excel spreadsheet Supplementary Table 4: on how well cpRNFLT predicts amnestic mild cognitive impairment (MCI) and amnestic mild neurocognitive disorder (NCD), respectively, per angular location.

Supplementary Table 4: Area under curve (AUC) in receiver operating characteristics analysis is presented for 360 degrees. This pointwise analysis is statistically significant at all 768 angular circumpapillary single locations (see Supplementary Figure 1). Sample B is highlighted in bold. We present the maximum AUC per sector (column D) and per location (column H) in the first columns, followed by the pointwise results. Investigation on how well cpRNFLT predicts MCI and mild NCD, respectively. Co-variates were accounted for as described in the methods section. Detailed results are shown in Supplementary Table 4 and Supplementary Figure 1. The RNFLT coefficients (Supplementary Table 1) and their corresponding AUC for our analysis (Supplementary Table 4) are followed by a multi-sector approach to establish AUC for a combination of regions (Figure 9). See also Supplementary Figures 2 to 13 for ROC curves for the retinal location with best and worst model performance.

Abbreviations: MCI, mild cognitive impairment; NCD, neurocognitive disorder

Supplementary Figure 1: **Prediction of amnestic MCI and amnestic mild NCD** Prediction of amnestic MCI and amnestic mild NCD from circumpapillary retinal nerve fibre layer thickness (cpRNFLT) by location.

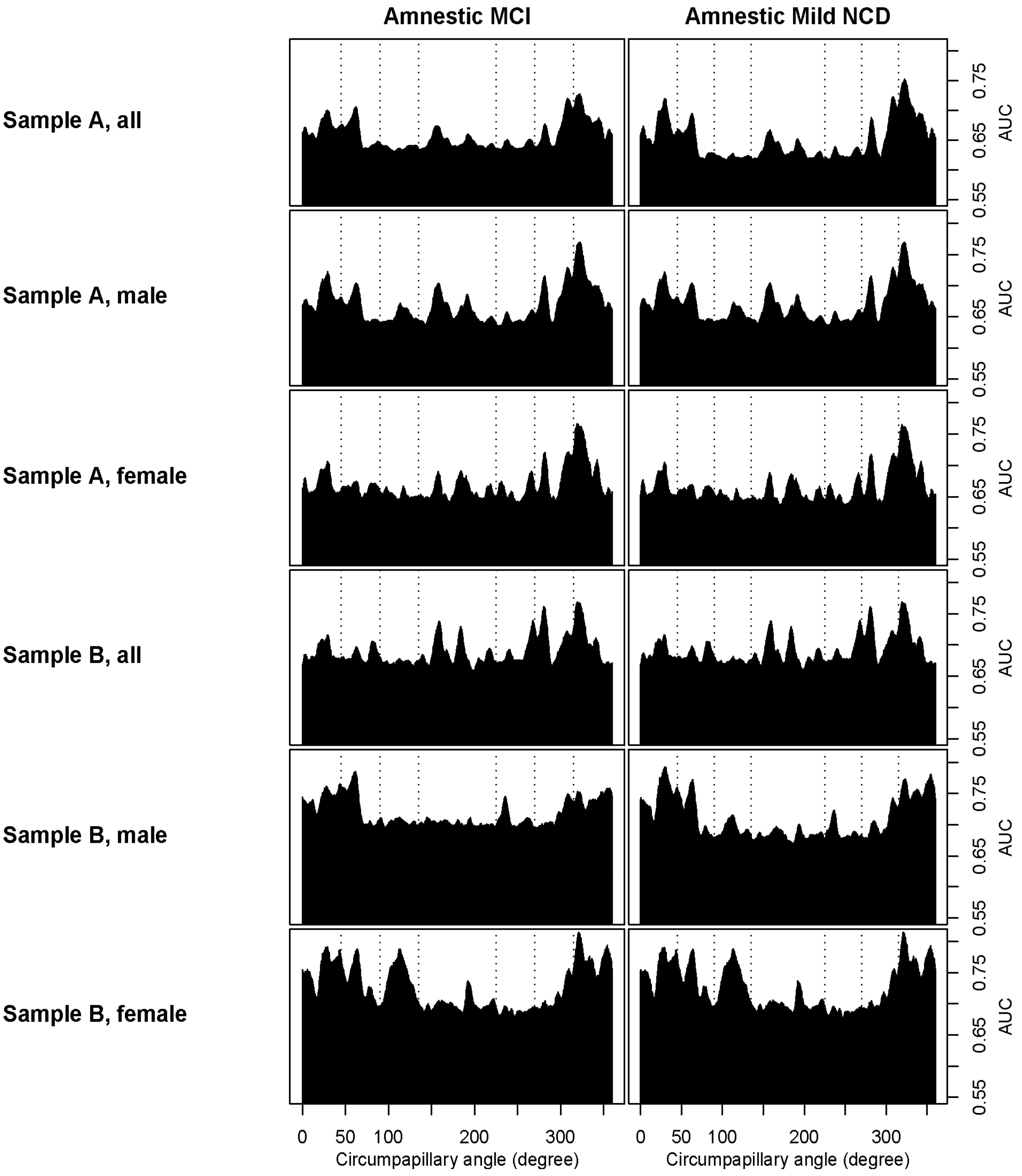

Supplementary Figure 1: Prediction of amnestic MCI and amnestic mild NCD from circumpapillary retinal nerve fibre layer thickness (cpRNFLT) by location. Area under curve (AUC) in receiver operating characteristics analysis is presented for 360 degrees. This pointwise analysis is statistically p-values (with cognitive domain as outcome) are shown in Supplementary Table 1. Higher numbers denote thicker cpRNFLT associated with better cognitive performance. We present significant at all 768 angular circumpapillary single locations.

The RNFLT coefficients (Supplementary Table 1) and their corresponding AUC for our analysis (Supplementary Table 4) are followed by a multi-sector approach to establish AUC for a combination of regions (Figure 9). One eye per participant entered analyses. Experimental unit: (uni-domain) amnestic MCI sample A/ sample B: N = 34 (f=19, m=15)/N = 25 (f=12, m=13); no MCI N =796 (f=317, m=479) / N = 658 (f=263, m=395), and (uni-domain) amnestic mild NCD: sample A/ sample B: N= 30 (f=15, m=15)/N = 25 (f=12, m=13); no NCD N = 816 (f=324, m=492) /N = 658 (f=263, m=395), see Table 1 for further information on sample sizes.

Abbreviations: MCI, mild cognitive impairment; NCD, neurocognitive disorder

Supplementary Figure 2 to Supplementary Figure 13: **ROC curves for the retinal location with best and worst model performance**

Full ROC curves for the retinal location with best and worst model performance, respectively, for each of the conditions for amnestic MCI and amnestic mild NCD. One eye per participant entered analyses. Experimental unit for (uni-domain) amnestic MCI: sample A: N = 34 eyes, No MCI= 796 eyes and (uni-domain) amnestic mild NCD: sample A: N = 30 eyes, no NCD: N = 816 eyes.

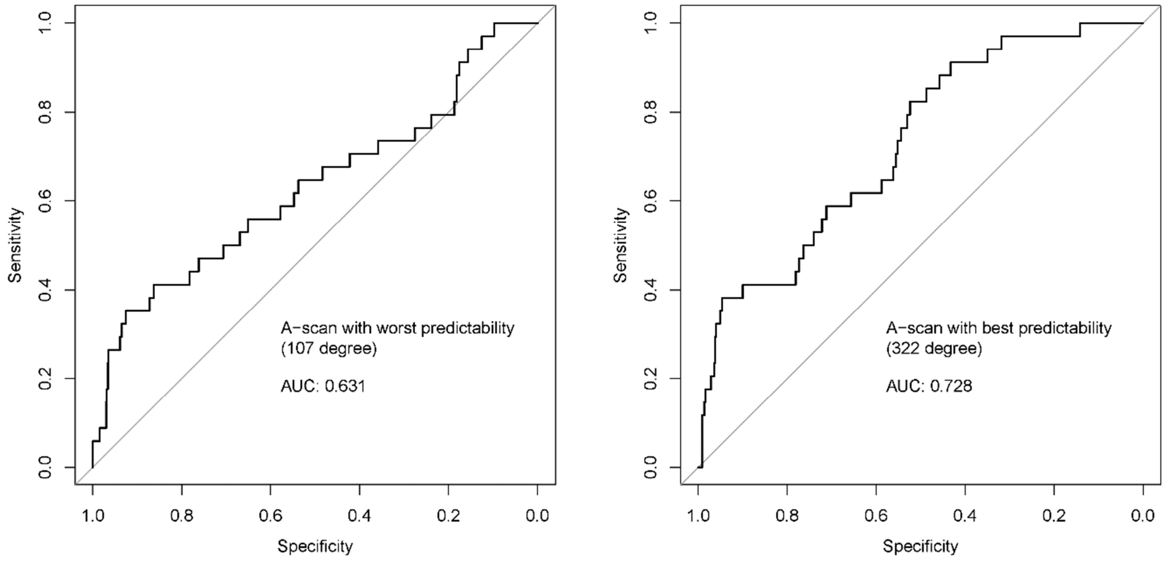

Supplementary Figure 2: Receiver operating characteristic (ROC) curves of models using the respective A-scan with worst (left hand side) and best (right hand side) predictability of amnestic MCI for all subjects in Sample A. Areas under the ROC curve (AUC) and the location of the respective A-scan on the circumpapillary B-scan in degree are provided on the bottom right part of each plot.

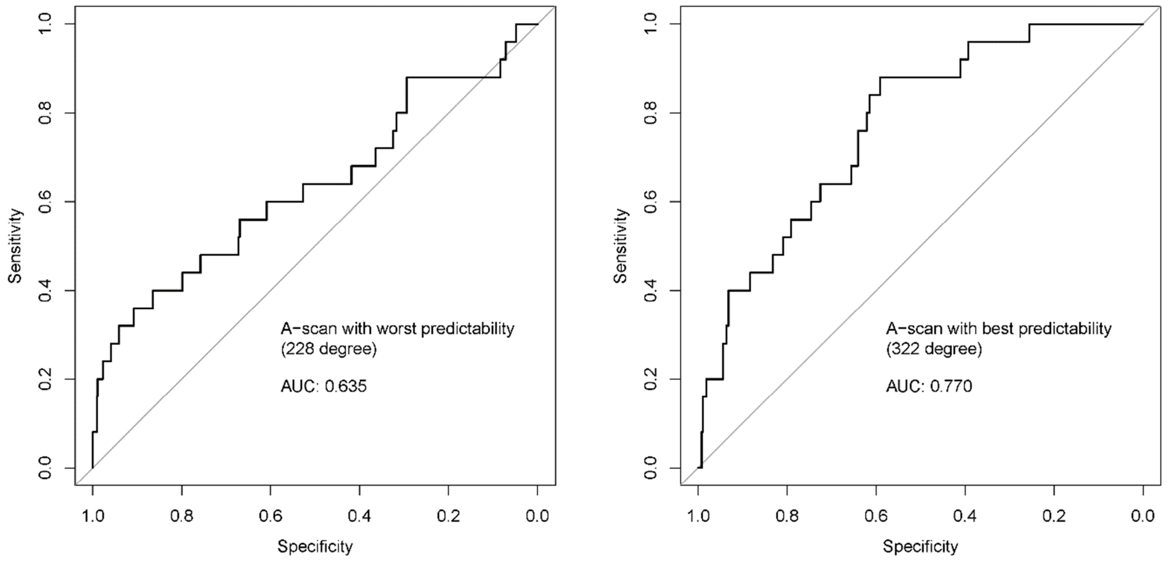

Supplementary Figure 3: Receiver operating characteristic (ROC) curves of models using the respective A-scan with worst (left hand side) and best (right hand side) predictability of amnestic MCI for all subjects in Sample B. Areas under the ROC curve (AUC) and the location of the respective A-scan on the circumpapillary B-scan in degree are provided on the bottom right part of each plot.

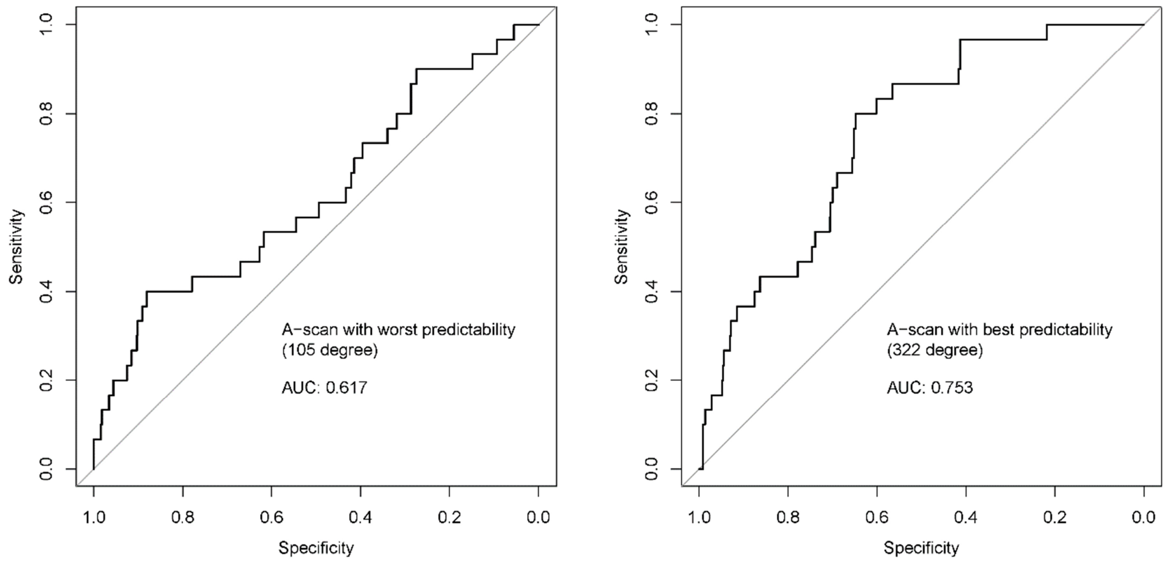

Supplementary Figure 4: Receiver operating characteristic (ROC) curves of models using the respective A-scan with worst (left hand side) and best (right hand side) predictability of amnestic mild NCD for all subjects in Sample A. Areas under the ROC curve (AUC) and the location of the respective A-scan on the circumpapillary B-scan in degree are provided on the bottom right part of each plot.

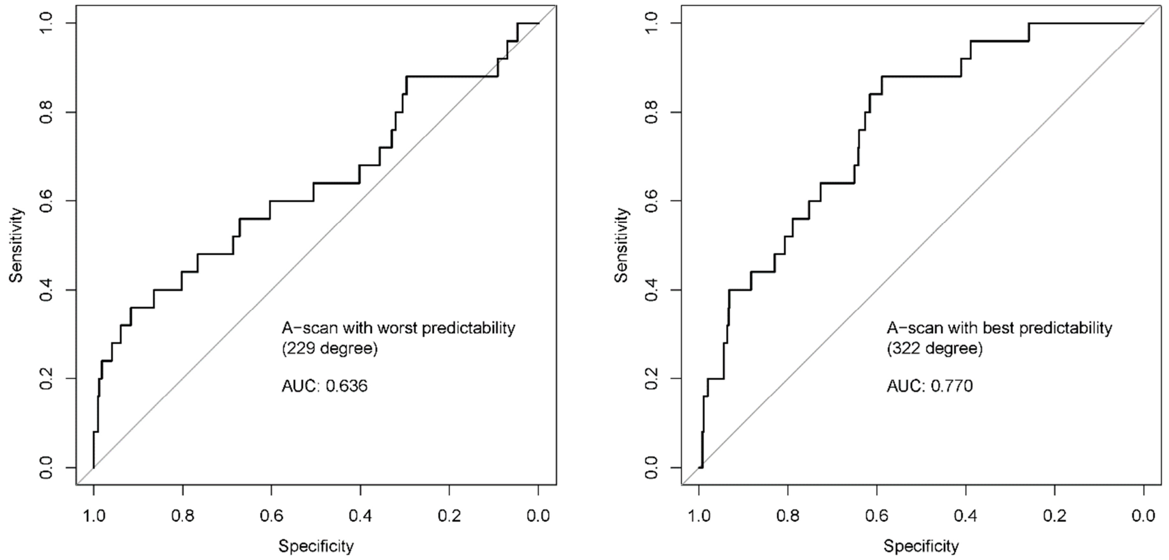

Supplementary Figure 5: Receiver operating characteristic (ROC) curves of models using the respective A-scan with worst (left hand side) and best (right hand side) predictability of amnestic mild NCD for all subjects in Sample B. Areas under the ROC curve (AUC) and the location of the respective A-scan on the circumpapillary B-scan in degree are provided on the bottom right part of each plot.

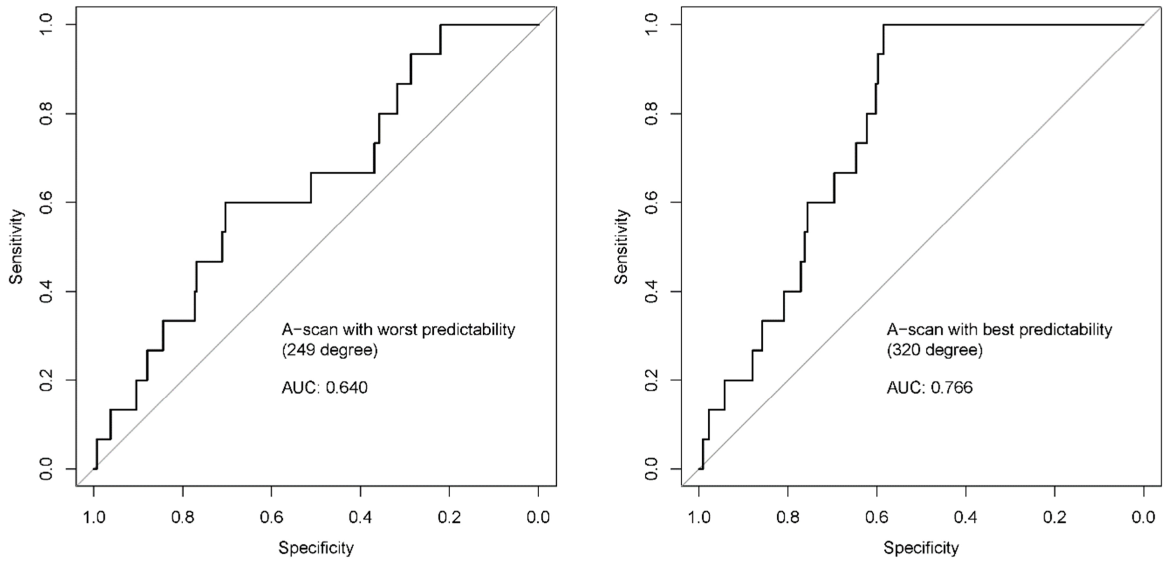

Supplementary Figure 6: Receiver operating characteristic (ROC) curves of models using the respective A-scan with worst (left hand side) and best (right hand side) predictability of amnestic MCI for men in Sample A. Areas under the ROC curve (AUC) and the location of the respective A-scan on the circumpapillary B-scan in degree are provided on the bottom right part of each plot.

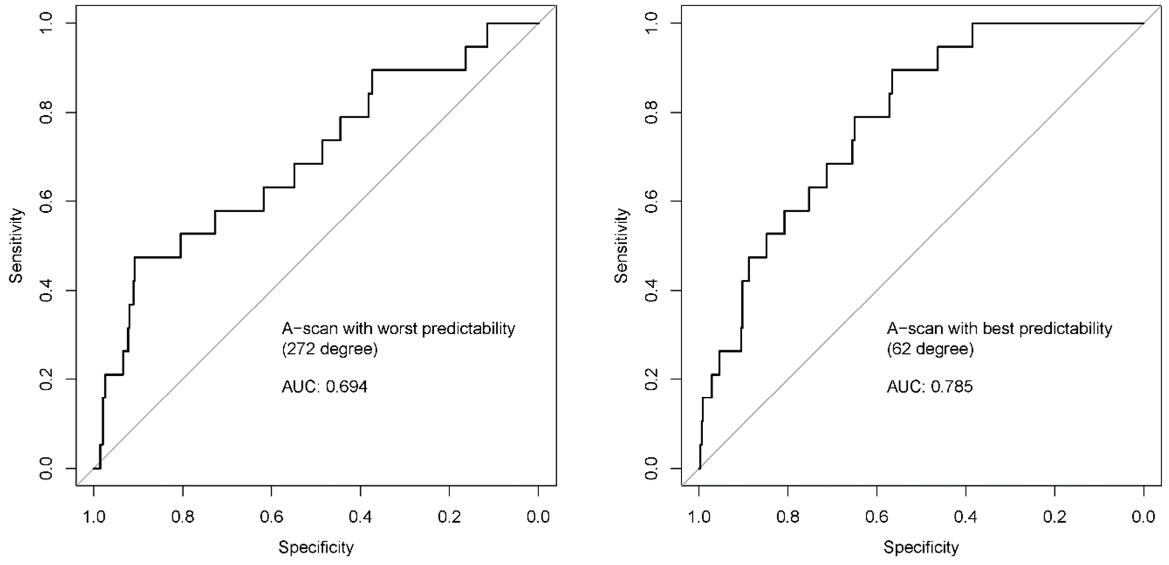

Supplementary Figure 7: Receiver operating characteristic (ROC) curves of models using the respective A-scan with worst (left hand side) and best (right hand side) predictability of amnestic MCI for women in Sample A. Areas under the ROC curve (AUC) and the location of the respective A-scan on the circumpapillary B-scan in degree are provided on the bottom right part of each plot.

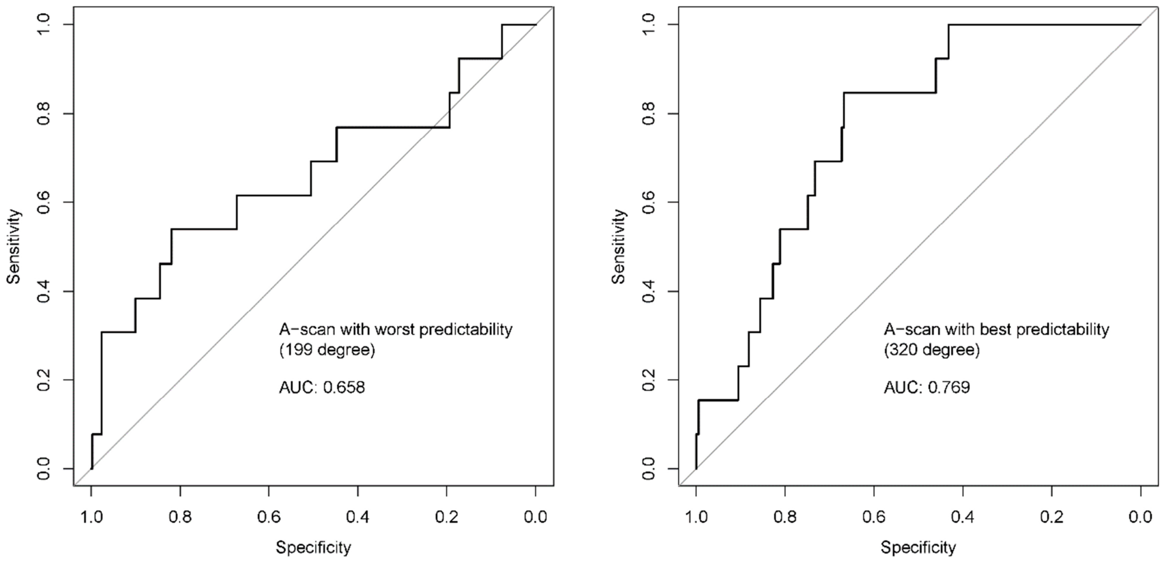

Supplementary Figure 8: Receiver operating characteristic (ROC) curves of models using the respective A-scan with worst (left hand side) and best (right hand side) predictability of amnestic MCI for men in Sample B. Areas under the ROC curve (AUC) and the location of the respective A-scan on the circumpapillary B-scan in degree are provided on the bottom right part of each plot.

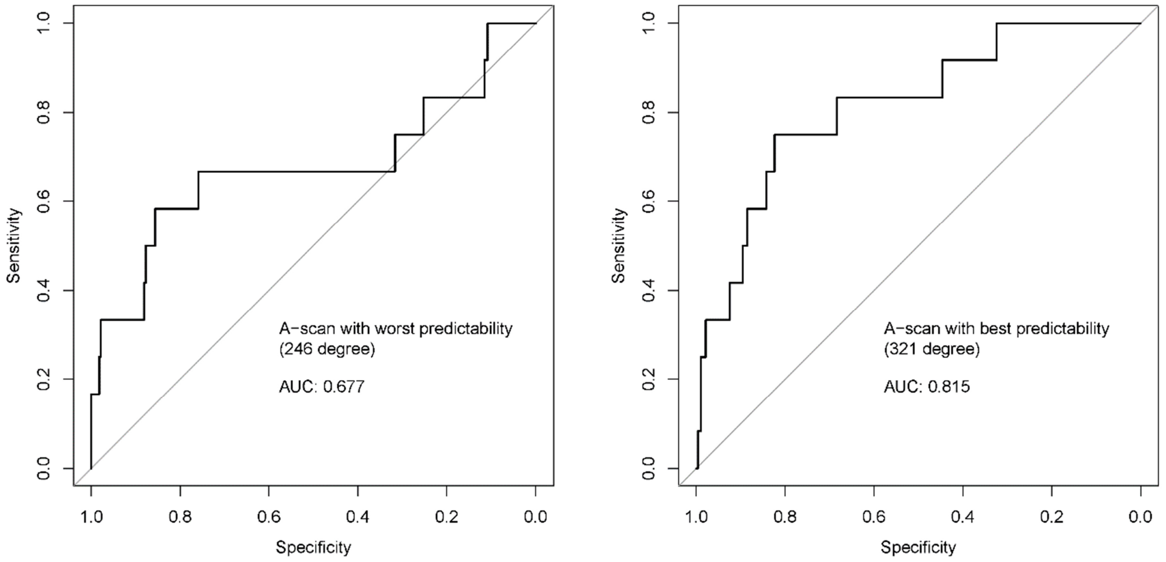

Supplementary Figure 9: Receiver operating characteristic (ROC) curves of models using the respective A-scan with worst (left hand side) and best (right hand side) predictability of amnestic MCI for women in Sample B. Areas under the ROC curve (AUC) and the location of the respective A-scan on the circumpapillary B-scan in degree are provided on the bottom right part of each plot.

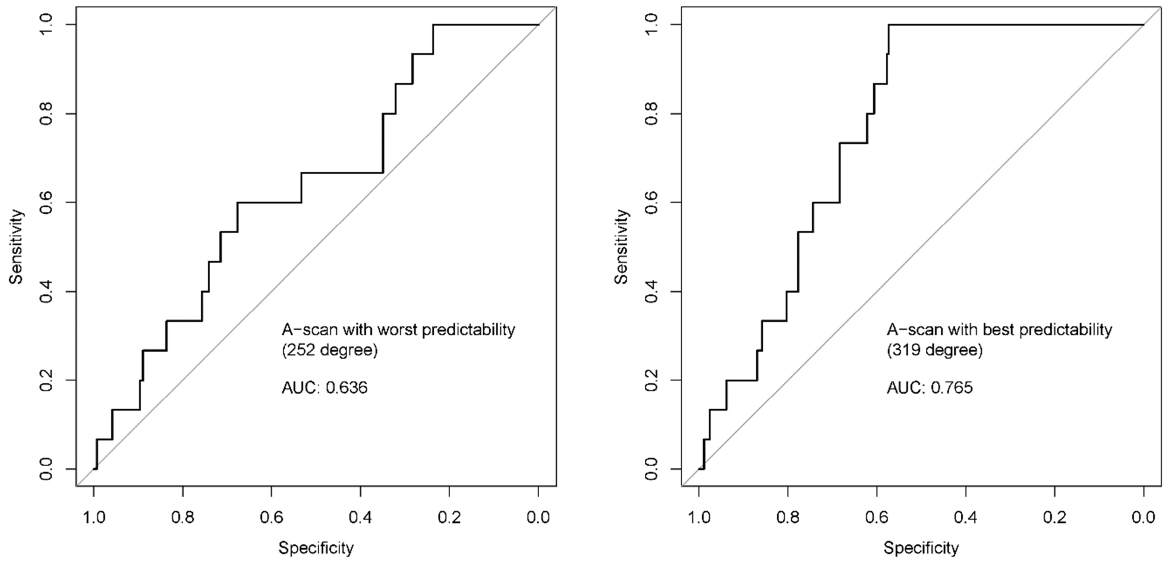

Supplementary Figure 10: Receiver operating characteristic (ROC) curves of models using the respective A-scan with worst (left hand side) and best (right hand side) predictability of amnestic mild NCD for men in Sample A. Areas under the ROC curve (AUC) and the location of the respective A-scan on the circumpapillary B-scan in degree are provided on the bottom right part of each plot.

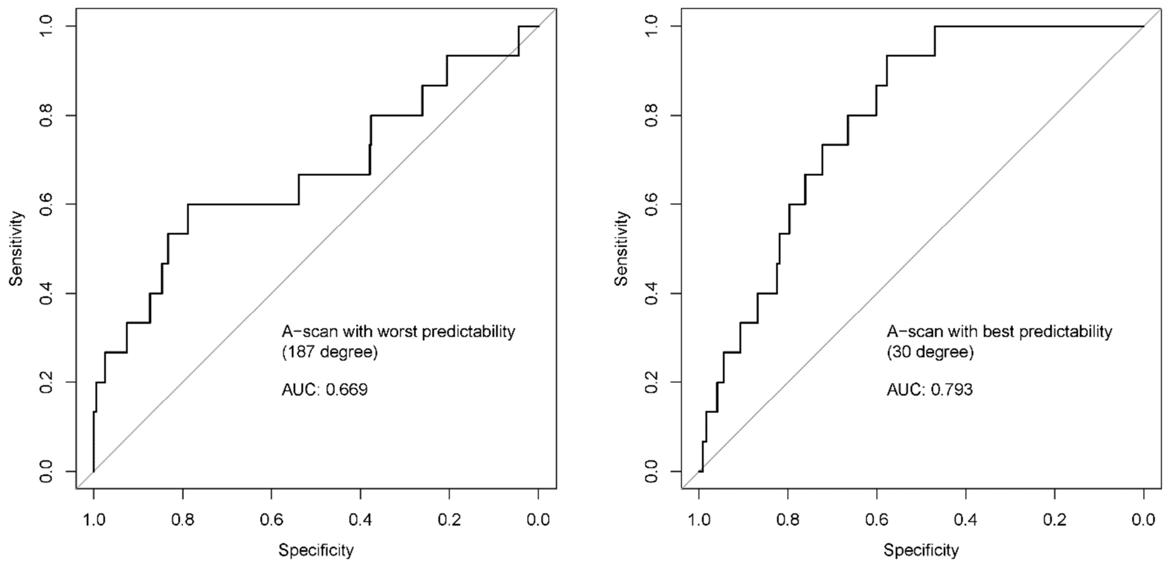

Supplementary Figure 11: Receiver operating characteristic (ROC) curves of models using the respective A-scan with worst (left hand side) and best (right hand side) predictability of amnestic mild NCD for women in Sample A. Areas under the ROC curve (AUC) and the location of the respective A-scan on the circumpapillary B-scan in degree are provided on the bottom right part of each plot.

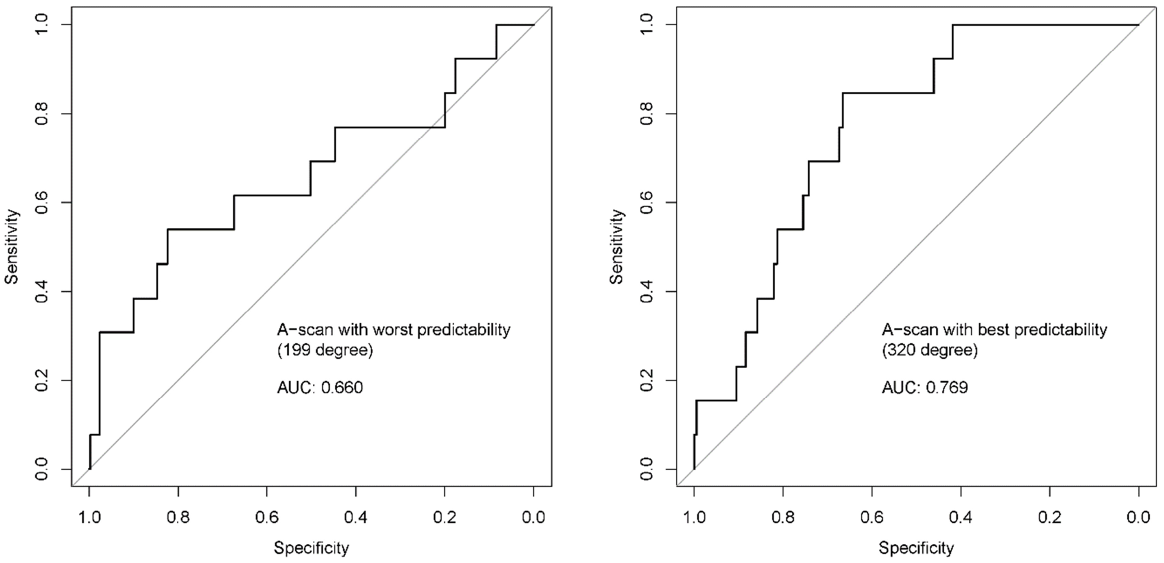

Supplementary Figure 12: Receiver operating characteristic (ROC) curves of models using the respective A-scan with worst (left hand side) and best (right hand side) predictability of amnestic mild NCD for men in Sample B. Areas under the ROC curve (AUC) and the location of the respective A-scan on the circumpapillary B-scan in degree are provided on the bottom right part of each plot.

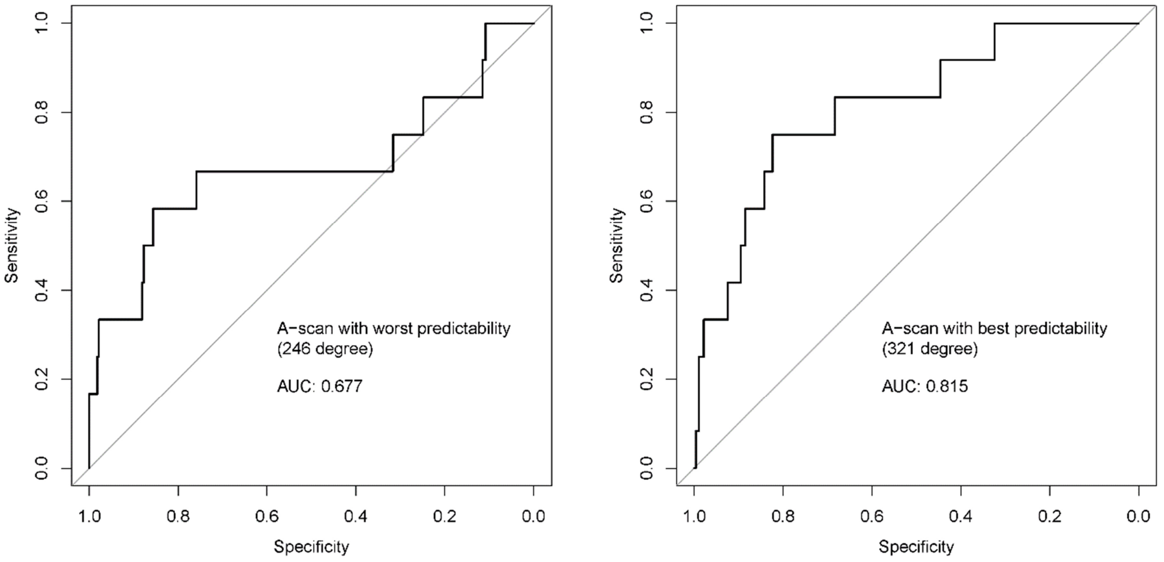

Supplementary Figure 13: Receiver operating characteristic (ROC) curves of models using the respective A-scan with worst (left hand side) and best (right hand side) predictability of amnestic mild NCD for women in Sample B. Areas under the ROC curve (AUC) and the location of the respective A-scan on the circumpapillary B-scan in degree are provided on the bottom right part of each plot.

Supplementary Figure 14: **Raw retinal nerve fibre layer thickness (cpRNFLT) in amnestic MCI**

Unadjusted circumpapillary retinal nerve fibre layer thickness in participants with amnestic MCI versus no MCI

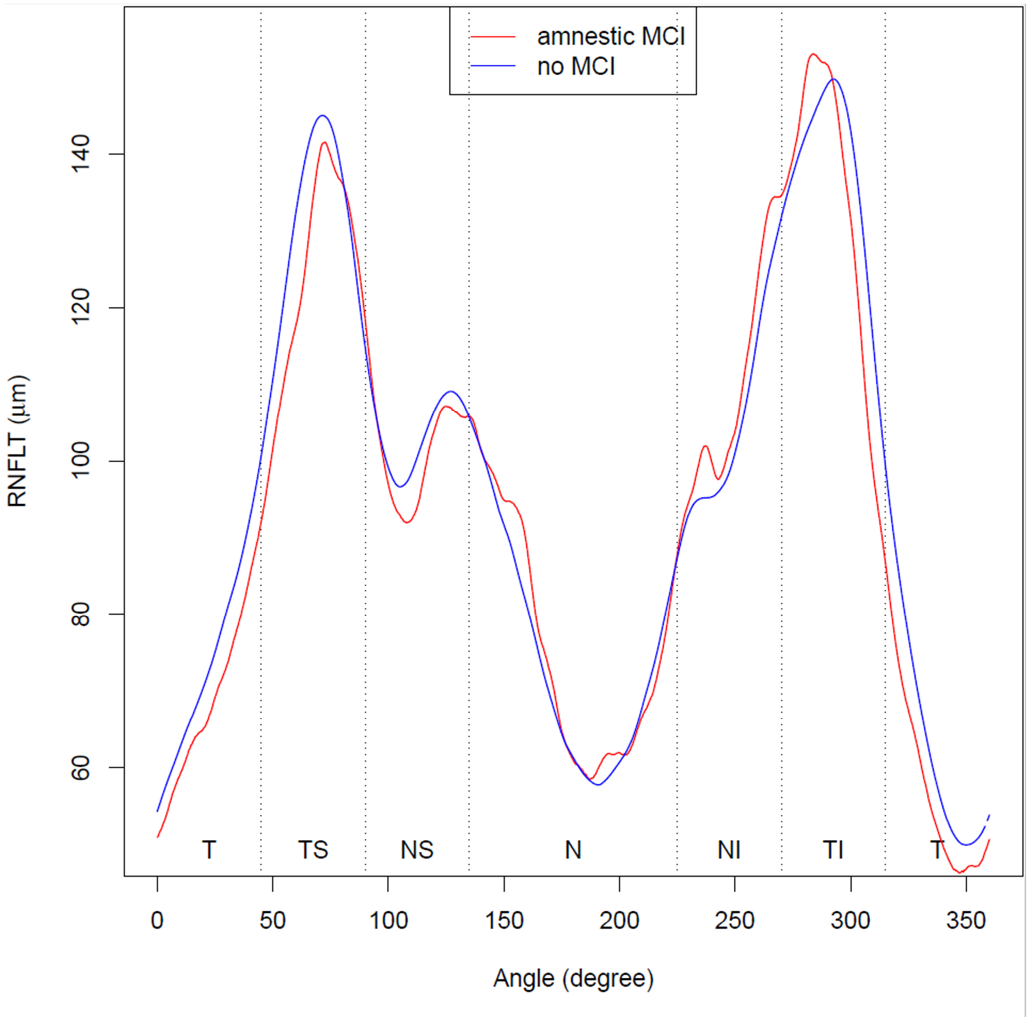

Supplementary Figure 14: In comparison to Larrosa et al. who investigated subjects with Alzheimer's disease (Larrosa et al. 2014), we plot circumpapillary retinal nerve fibre layer thickness (cpRNFLT) for 360 degrees by pointwise analysis for 768 specific locations to compare uni-domain amnestic mild cognitive impairment (MCI) and no MCI. The TSNIT (temporal – superior – nasal – inferior – temporal) plot across all cpRNFLT measurement points on the circle highlights the specific locations where cpRNFLT thickness differences are present for amnestic mild cognitive impairment. However, adjustment is key before presenting associations of cpRNFLT and cognition. The TSNIT graph presents raw data without our standard procedure to implement strict adjustment for potential confounders. When comparing this raw data to our Figure 7, one can see that co-variables (e.g. age, radius) explain much of the variance present, which we have adjusted for in our analysis presented in the main text. One eye per participant entered analyses. Experimental unit for (uni-domain) amnestic MCI: sample A: N = 34 eyes, No MCI= 796 eyes and (uni-domain) amnestic mild NCD: sample A: N = 30 eyes, no NCD N = 816 eyes.

Supplementary Figure 15: **Raw retinal nerve fibre layer thickness (cpRNFLT) in amnestic mild NCD**

Unadjusted circumpapillary retinal nerve fibre layer thickness in participants with amnestic mild NCD versus no NCD

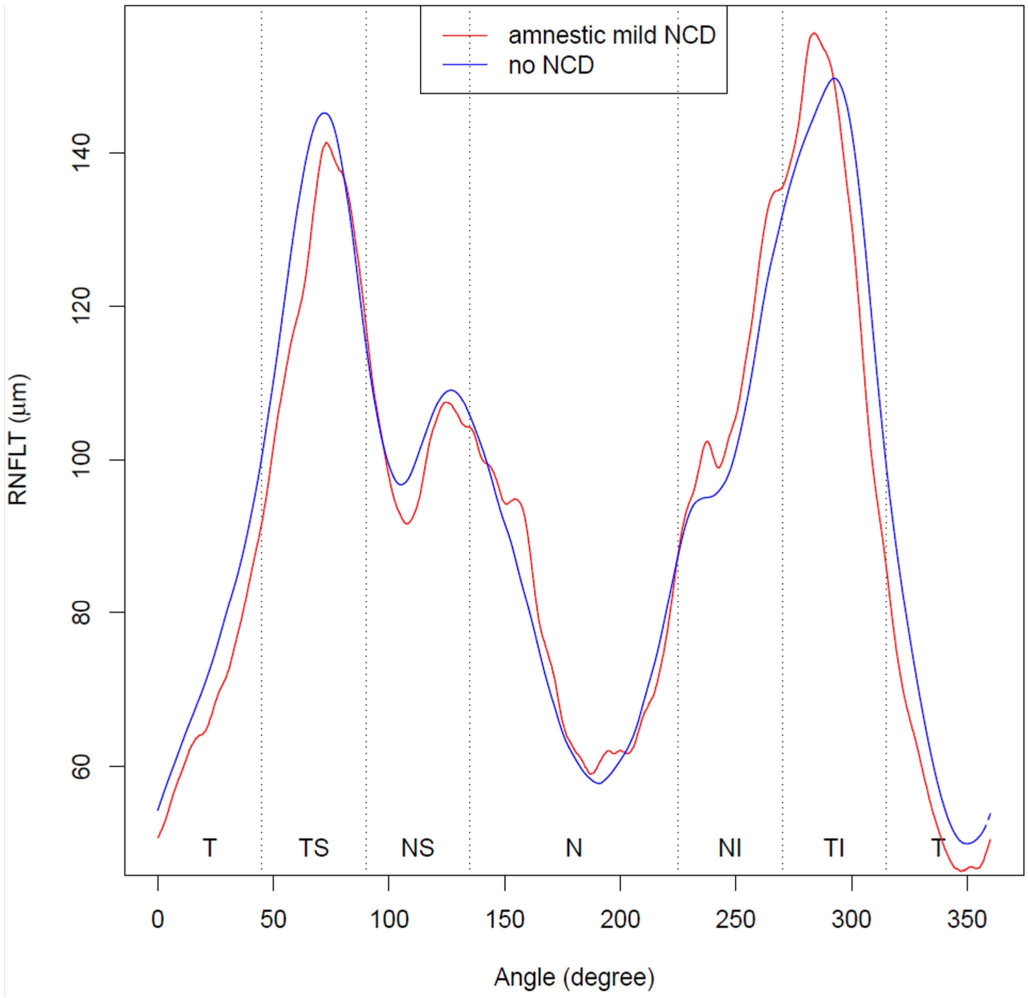

Supplementary Figure 15: In comparison to Larrosa et al. who investigated subjects with Alzheimer's disease (Larrosa et al. 2014), we plot circumpapillary retinal nerve fibre layer thickness (cpRNFLT) for 360 degrees by pointwise analysis for 768 specific locations to compare amnestic mild neurocognitive disorder (NCD) and no NCD. The TSNIT (temporal – superior – nasal – inferior – temporal) plot across all cpRNFLT measurement points on the circle highlights the specific locations where cpRNFLT thickness differences are present for amnestic mild cognitive impairment. However, adjustment is key before presenting associations of cpRNFLT and cognition. The TSNIT graph presents raw data without our standard procedure to implement strict adjustment for potential confounders. When comparing this raw data to our Figure 7, one can see that co-variables (e.g. age, radius) explain much of the variance present, which we have adjusted for in our analysis presented in the main text. One eye per participant entered analyses. Experimental unit for (uni-domain) amnestic MCI: sample A: N = 34 eyes, No MCI= 796 eyes and (uni-domain) amnestic mild NCD: sample A: N = 30 eyes, no NCD N = 816 eyes.

Supplementary Figure 16: **An example comparison of participant with normal cognition (Figure 2: A, C) and participant with amnestic mild cognitive impairment (MCI) / amnestic mild neurocognitive disorder (NCD) (Figure 2: B, D).**

Raw data for circumpapillary retinal nerve fibre layer thickness in participant with normal cognition.

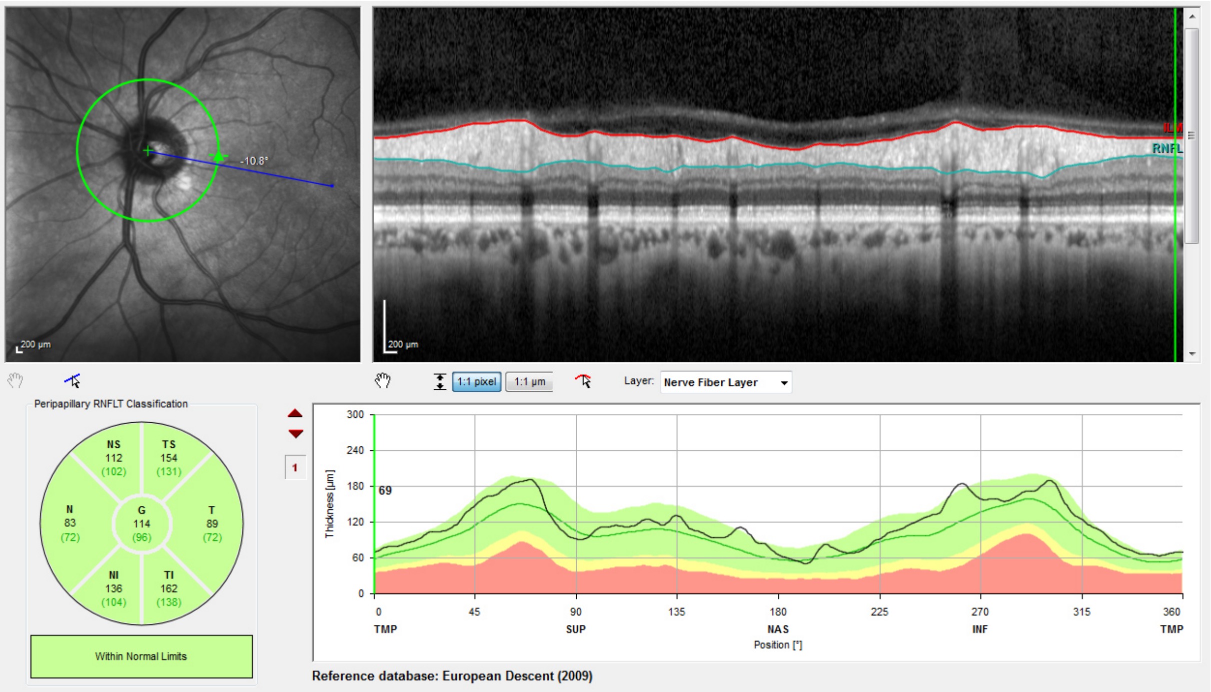

Supplementary Figure 16: An example comparison of participant with normal cognition (Figure 2: A, C) and participant with amnestic mild cognitive impairment (MCI) / amnestic mild neurocognitive disorder (NCD) (Figure 2: B, D). High-resolution T1-weighted anatomical MR images (Figure 2: A,B; voxel resolution = 1x1x1mm3) showing coronal slices of the brain with specific focus on the hippocampus region (inset). Note mesial temporal lobe atrophy (arrow) and temporoparietal cortical atrophy of image B compared to image A. Optical coherence tomography (OCT)-determined circumpapillary retinal nerve fibre layer thickness (cpRNFLT; Figure 2: C,D). The green circle depicts the location of the measurement around the optic nerve head (ONH) in a fundus image of the left eye. Each clockwise measurement starts temporally. The right OCT-image depicts the B-scan where cpRNFL is located between the red and the blue segmentation lines. Extended information for both OCT-derived images is presented in Supplementary Figure 16 for the participant with normal cognition, or in Supplementary Figure 17 for the participant with amnestic mild cognitive impairment (MCI) / amnestic mild neurocognitive disorder (NCD).

Here the bottom right image illustrates a printout of the Spectralis spectral domain OCT cpRNFLT measurement (black line) depicted on the device-based normative data set (not utilized in this study). Measurements within the green shaded area display the 5th to the 95th percentile of the device-based normative set (n=201), yellow indicates the 1st to 5th percentile and red areas depict below the 1st percentile. Note that this section of the machine-printout does not take into account covariates (e.g. age, sex, refraction). Interestingly, in Supplementary Figure 17, the black line of the amnestic MCI / amnestic mild NCD participant displays much thinner cpRNFLT throughout compared to the cognitive healthy participant in Supplementary Figure 16. The six sector values (bottom left image) highlight this. Note: both example participants are part of examined study samples A and B.

Abbreviations: MCI, mild cognitive impairment; NCD, neurocognitive disorder

Supplementary Figure 17: **An example comparison of participant with normal cognition (Figure 2: A, C) and participant with amnestic mild cognitive impairment (MCI) / amnestic mild neurocognitive disorder (NCD) (Figure 2: B, D).**

Raw data for circumpapillary retinal nerve fibre layer thickness in participant with amnestic mild cognitive impairment (MCI) / amnestic mild neurocognitive disorder (NCD).

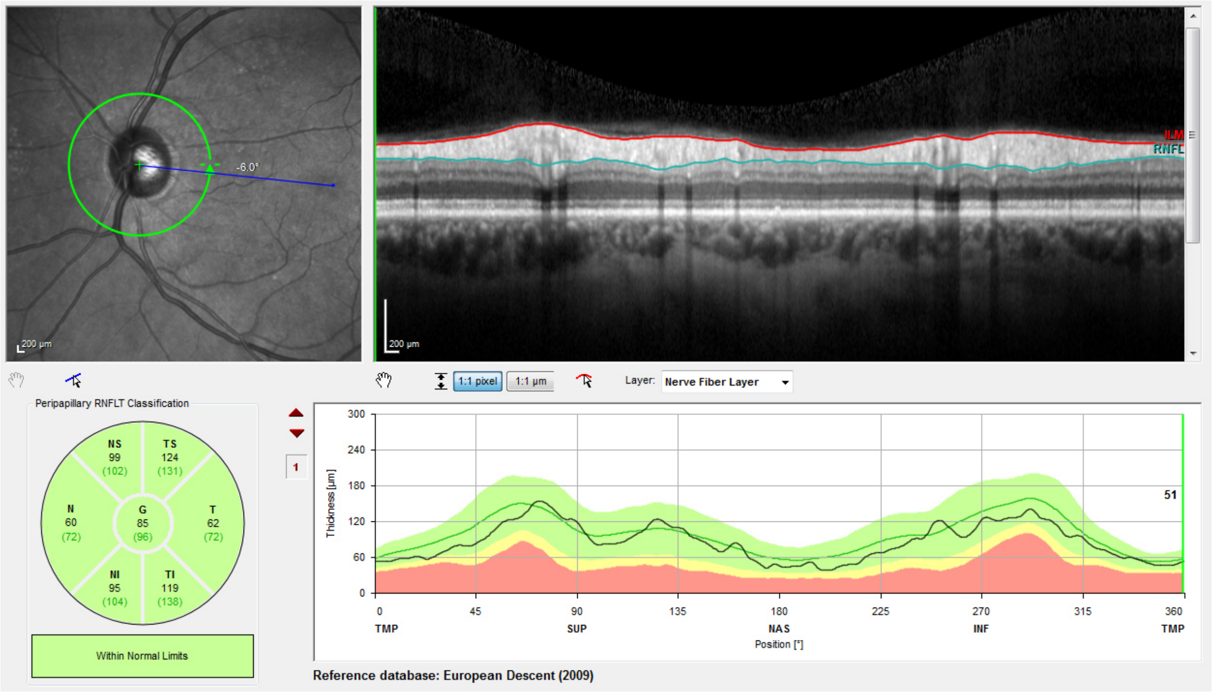

Supplementary Figure 17: An example comparison of participant with normal cognition (Figure 2: A, C) and participant with amnestic mild cognitive impairment (MCI) / amnestic mild neurocognitive disorder (NCD) (Figure 2: B, D). High-resolution T1-weighted anatomical MR images (Figure 2: A,B; voxel resolution = 1x1x1mm3) showing coronal slices of the brain with specific focus on the hippocampus region (inset). Note mesial temporal lobe atrophy (arrow) and temporoparietal cortical atrophy of image B compared to image A. Optical coherence tomography (OCT)-determined circumpapillary retinal nerve fibre layer thickness (cpRNFLT; Figure 2: C,D). The green circle depicts the location of the measurement around the optic nerve head (ONH) in a fundus image of the left eye. Each clockwise measurement starts temporally. The right OCT-image depicts the B-scan where cpRNFL is located between the red and the blue segmentation lines. Extended information for both OCT-derived images is presented in Supplementary Figure 16 for the participant with normal cognition, or in Supplementary Figure 17 for the participant with amnestic mild cognitive impairment (MCI) / amnestic mild neurocognitive disorder (NCD).

Here the bottom right image illustrates a printout of the Spectralis spectral domain OCT cpRNFLT measurement (black line) depicted on the device-based normative data set (not utilized in this study). Measurements within the green shaded area display the 5th to the 95th percentile of the device-based normative set (n=201), yellow indicates the 1st to 5th percentile and red areas depict below the 1st percentile. Note that this section of the machine-printout does not take into account covariates (e.g. age, sex, refraction). Interestingly, in Supplementary Figure 17, the black line of the amnestic MCI / amnestic mild NCD participant displays much thinner cpRNFLT throughout compared to the cognitive healthy participant in Supplementary Figure 16. The six sector values (bottom left image) highlight this. Note: both example participants are part of examined study samples A and B.

Abbreviations: MCI, mild cognitive impairment; NCD, neurocognitive disorder

Supplementary References

Larrosa, Jose M.; Garcia-Martin, Elena; Bambo, Maria P.; Pinilla, Juan; Polo, Vicente; Otin, Sofia et al. (2014): Potential new diagnostic tool for Alzheimer's disease using a linear discriminant function for Fourier domain optical coherence tomography. In: *Invest. Ophthalmol. Vis. Sci.* 55 (5), S. 3043–3051. DOI: 10.1167/iovs.13-13629.
