# Supplementary figures and images for: Retinal nerve fibre layer thickness is associated with attention and predicts risk states of dementia
Source: Brain Commun. 2025 Dec 5;7(6):fcaf464. doi: 10.1093/braincomms/fcaf464 (PMC12679710; doi:10.1093/braincomms/fcaf464)

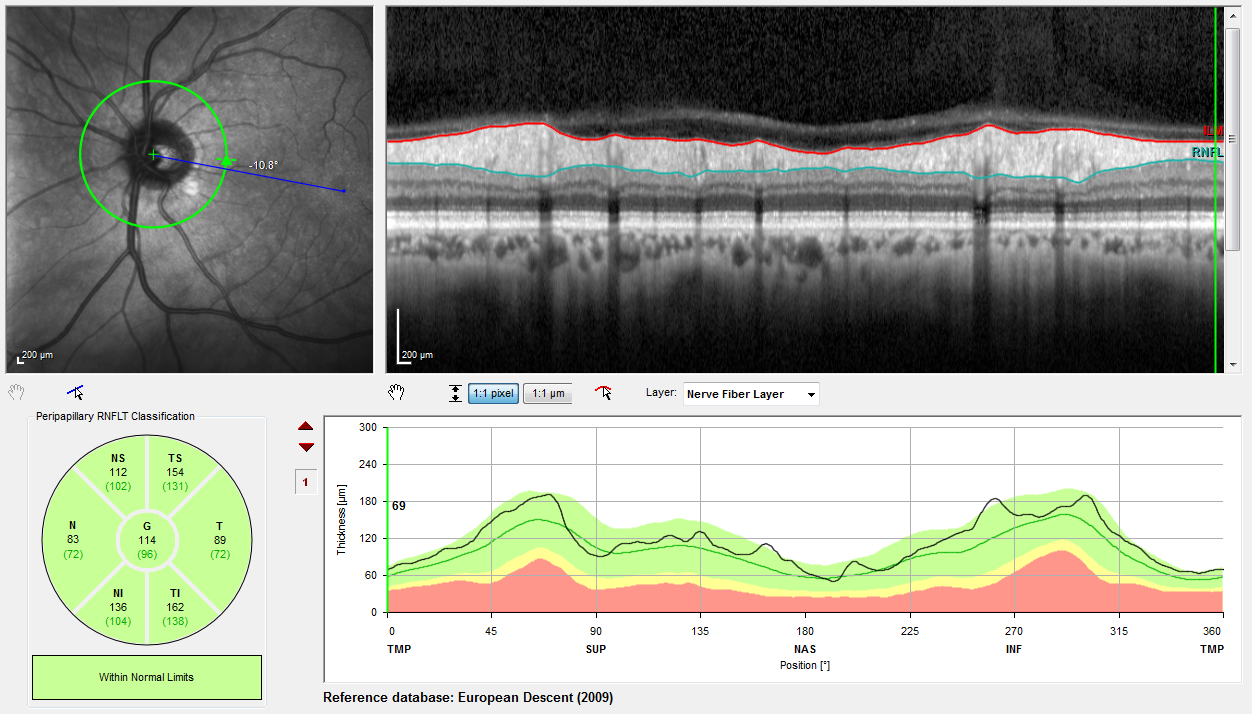

Supplement: fcaf464_Supplementary_Data [file fcaf464_supplementary_data.zip › supplementary_figure_16_healthy_OS.tif]

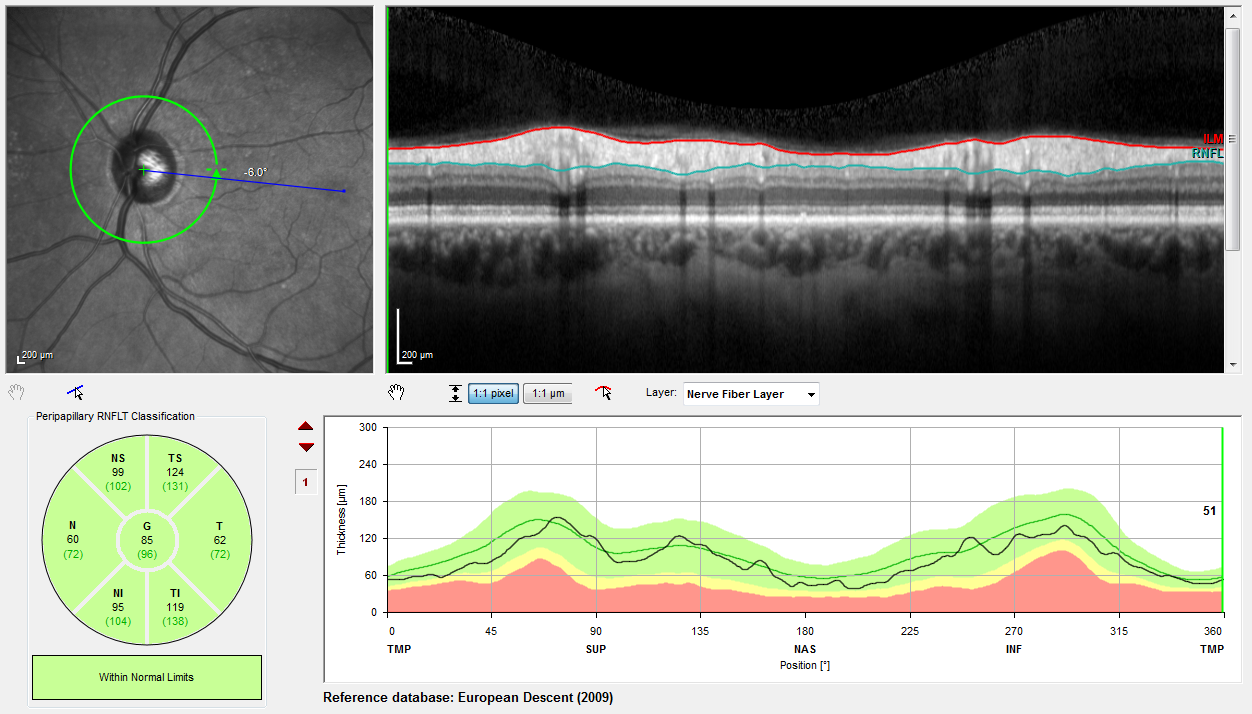

Supplement: fcaf464_Supplementary_Data [file fcaf464_supplementary_data.zip › supplementary_figure_17_amnesticMCI+mNCD_OS.tif]
